# Clinical significance of low expression of CADM3 in breast cancer and preliminary exploration of related mechanisms

**DOI:** 10.1186/s12885-024-12114-y

**Published:** 2024-03-21

**Authors:** Huiyang Ren, Zhen Wang, Lei Zhang, Guolian Zhu, Feng Li, Bo Chen

**Affiliations:** 1 Department of Breast Surgery, the First Hospital of China Medical University, 155 Nanjing North Street, Heping District, Shenyang City, Liaoning 110001 China; 2 Department of Cell Biology, Key Laboratory of Cell Biology of National Health Commission of the PRC, Key Laboratory of Medical Cell Biology of Ministry of Education of the PRC, China Medical University, No.77, Puhe Road, Shenyang, Liaoning 110122 China; 3 Department of Breast Surgery, the Fifth People’s Hospital of Shenyang, 188 Xingshun Street, Tiexi District, Shenyang City, Liaoning 110023 China

**Keywords:** CADM3, Breast cancer, Clinic pathological features, Proliferation, Migration, MAPK pathway

## Abstract

**Background:**

Cell adhesion molecule 3 (CADM3), a transmembrane glycoprotein on cell membranes, plays a role in the way of ligand and receptor interaction. However, there are few studies on CADM3 in tumors, and how it works in breast cancer (BC) remains unclear.

**Methods:**

The Cancer Genome Atlas (TCGA) database and clinical samples were used to analyze CADM3 expression and its correlation with clinicopathological factors and prognosis. Its correlation with immune infiltration was analyzed by TCGA. The effects of CADM3 on proliferation and migration were investigated by cell clonal formation, CCK-8, cell scratch and transwell assay. Protein interaction network was prepared and the function prediction of related genes was conducted. The correlation between CADM3 and MAPK pathway was further explored by western blot experiment.

**Results:**

The expression of CADM3 in BC tissues were significantly lower than that in adjacent normal tissues. High level of CADM3 was related to better prognosis of BC patients. CADM3 was an independent prognostic factor for BC. Expression of CADM3 was significantly associated with the status of ER and PR, age and PAM50 subtypes. CADM3 positively related to many immune infiltrating cells. Overexpression of CADM3 can notably reduce cell proliferation and migration. CADM3 was related to MAPK pathway and the phosphorylation of ERK1/2 and JNK1 was inhibited in BC cells with high CADM3.

**Conclusions:**

Our research reveals the clinical significance of CADM3 in BC and indicates the critical roles of CADM3 in immune infiltration and MAPK pathway.

**Supplementary Information:**

The online version contains supplementary material available at 10.1186/s12885-024-12114-y.

## Introduction

Breast cancer (BC) ranks first in female malignant tumors and is also the main cause of female cancer mortality. According to the global cancer statistics in 2020, BC has overtaken lung cancer as the most common cancer in women [1]. At present, the conventional treatment methods of BC include surgery, chemotherapy and radiotherapy, which have greatly improved the treatment effect and the prognosis of BC patients [2]. Although the survival rate of BC patients has been significantly improved, how to inhibit the development of BC and reduce the chance of local recurrence and metastasis is still one of the key issues. Therefore, the exploration of specific molecular markers for the treatment of BC has become a research hotspot, which is conducive to the early diagnosis and prognosis of patients.

Cellular adhesion molecules (CAMs) are proteins located on the membrane of biological cells, which participate in intercellular recognition, cell activation and signal transduction in the process of cell movement, proliferation and apoptosis. They fall into four main categories: integrins, selectors, cadherin family and immunoglobulin family [3]. Among them, the immunoglobulin family plays an crucial part in the mutual recognition between cells [4, 5], and the recently discovered CADMs family, belonging to one of them, acts as transmembrane glycoproteins in a variety of tissues in the form of cell adhesion molecules. Recent studies have shown that CADMs family members are associated with the occurrence and development of tumors, and they are low expressed in various tumors [6–10]. CADMs molecules are of critical importance in tumor development and metastasis by regulating the proliferation, inducing apoptosis of tumor cells and changing the growth characteristics of tumor cells. In addition, CADMs has a certain correlation with immune infiltrating cells, and some studies have shown that the silencing of CADMs molecules may be related to the mechanism of tumor immune evasion [11–13]. CADM3, also known as BIgR, FLJ10698, IGSF4B, NECL1, Necl-1, SynCAM3, TSLL1, located on chromosomes 1q21.2 to q22, encodes a protein consisting of 398 amino acids. It is an immunoglobin-like cell adhesion molecule specific for neural tissues [14]. At present, there are few studies on CADM3 in tumors, and there is no related report in BC.

Our research first analyzed the expression and prognostic value of CADM3 in using clinical breast cancer samples from patients and multiple bioinformatics databases. We then examined the association between CADM3 and clinical features. Next, we verified the effect of CADM3 on proliferation and migration in BC. Protein-protein interaction (PPI) networks were produced. This study demonstrated the associations of CADM3 with clinical features, immune infiltration, and MAPK pathway in BC. In conclusion, the upregulation of CADM3 was correlated with favorable prognosis, immune infiltration and MAPK pathway in breast cancer patients.

## Methods and materials

### Database and correlation analysis

We used The Cancer Genome Altas (TCGA) (https://portal.gdc.cancer.gov/) to do pan-cancer expression analysis. RNAseq data of invasive breast carcer (TCGA-BRCA) were downloaded and collated from the TCGA database and extracted in TPM format. Data is processed and converted using Perl scripts. “ggplot2[3.3.6]”, “stats [4.2.1]” and “car” package were used to analyze the paired and unpaired differential expression of the data. The statistical method of pan-cancer analysis and the unpaired differential expression was Wilcoxon rank sum test. The statistical method of the unpaired differential expression was Wilcoxon signed rank test. Using DESeq2 [1.36.0], edgeR [3.38.2] package for CADM3 single genetic differences, and set up the conditions of *P* < 0.05 and| log FC| > 2 to further screening.

The R package “ggplot2[3.3.6]” “survminer”, and “survival [3.3.1]” were applied to analyze the patient survival data in TCGA database. The log-rank test was used for all the survival analysis.

Clinical sample data were downloaded from the TCGA database to analysis the differential expression of CADM3. ER, PR, HER2 expression status, age, TNM stage, and PAM50 subtypes were selected as analysis factors. And the data were analyzed by R [4.2.1] and R package “ggplot2 [3.3.6]”. Wilcoxon rank sum test was used for statistical analysis of ER, PR,HER2 status and age group. Kruskal-Wallis Test and Dunn’s test were used for the PAM50 group.

R package “DESeq2 (version 1.26.0)” was used to filter differentially expressed genes (DEGs) [15, 16] (p.adj < 0.05,|log2FoldChange|>2) between high expression group and low expression group of CADM3 divided by the median value in TCGA database. The R package “ggplot2 (version 3.3.6)” was used to plot the volcano figure and the heatmap. The statistical method of the heatmap was Spearman.

The enrichment score was defined by the single sample GSEA to represent the absolute enrichment degree of the gene set in each sample within a given dataset of the R package “GSVA” [17]. We also calculated the standardized enrichment score for each immune category. And we obtained the gene set features of various immune cells from the previous study [18].

We used STRING (https://string-db.org) [19] to determine the interactive protein of CADM3. We constructed the PPI network using Cytoscape software and CytoHubba [20] and identified the first 15 central genes. In addition, the function and mechanism of selected hub genes were predicted by GeneMANIA (https://genemania.org/), which is a flexible user-friendly web site for generating hypotheses about gene function, analyzing gene lists and prioritizing genes for functional assays [21].

### Patients and samples

We collected 40 pairs of fresh BC tissues and corresponding adjacent normal tissues surgically removed in 2022, which were transported and preserved in liquid nitrogen tanks as soon as possible after surgical removal for detection of mRNA and protein levels. Paraffin sections of BC tissues from 325 patients surgically removed in 2015 were collected and stored at 4℃ in the dark for immunohistochemical experiments. This study was approved by Ethics Committee of the First Hospital of China Medical University (Number: AF-SOP-07-1.1-01).

### RNA transfection

CADM3 overexpression and the negative control lentivirus were bought from Shanghai Genechem Co., LTD Cell planking was performed before transfection, and cell density was determined according to cell growth rate and shape, so that cell coverage reached about 30% on the day of transfection. The number of virus particles was calculated according to the MOI value, and the virus stock solution was diluted. ​The dilute solution of the virus was added to a complete medium free of antibiotics. The culture medium was changed after 24 h in the incubator, and then the medium was changed according to the state of the cells.The sequences of CADM3 and its control for RNA transfection can be seen in supplementary Table 1.

### Antibodies

The antibodies used in the experiment are as follows: CADM3 (Affinity, #DF3537); GAPDH (protein tech, #60004-1-Ig); CD3 (Abmart, #T55982); CD8 (Abmart, #T40010); p38 (Abmart, #T40075); Phospho-p38(Thr180/Tyr182) (Abmart, #T40076); ERK1/2 (Abmart, #T55487); Phospho-Erk1(T202/Y204) + Erk2(T185/Y185) (Abmart, #T40072); JNK1/2/3 (Abmart, #T40073); p-JNK1/2/3(Thr183 + Tyr185) (Abmart, #T40074).

### Western blot analysis

Cells were lysed for 15 min at 4℃ using an ice cold RIPA buffer. Through a series of operations like sonication and centrifugation, we obtained the total cell extract. Put the cell extract into boiled water for 5 min to make it denatured. Put the protein into SDS–PAGE (10% gels), 30 micrograms per well, and then transferred onto a PVDF membrane. 1 h sealed in 5% milk powder solution, then added diluted primary antibody and incubated overnight at 4℃. The secondary antibody was incubated for 1 h at 25℃. ECL system was used for detecting the samples.

### Real time PCR

RNA from tissues and cells was extracted by TRIzol reagent and then cDNA synthesis was performed. Each tubule was added with 10µL SYBR, 8.2µL ddH2O, 0.8µL primer and 1µL template for quantitative real-time PCR. Forty cycles were repeated at 50°C for 2min, 95°C for 10min, 95°C for 15s, and 60°C for 1min. Relative expression levels were calculated by 2-ΔΔCt and standardized by GAPDH. The sequences of the primer pairs are shown below: CADM3 forward, 5’-CTCGGTGACATTCCAGGTTACC-3’, reverse, 5’-GCCTAATCATCGCAGTTGGTG TG-3’; GAPDH forward, 5’-GGAGCGAGATCCCTCCAAAAT-3’, reverse, 5’-GCTGTTGTCATACTTCTCATGGG-3’.

### Immunohistochemistry

The tissue sections were deparaffinized and rehydrated, then citrate buffer was used to perform antigen retrieval. Next, the endogenous peroxidase activity was blocked by 3% H_2_O_2_. Then, incubated the sections with the CADM3 antibody (Affinity, #DF3537, 1:100) overnight at 4 centigrade. After that, the secondary antibodies incubation, DAB regents (Maxim, DAB-0031/1031) staining, and hematoxylin counterstaining were performed. The CADM3 expression score was measured by multiplying the intensity score of staining (negative, 0; weak, 1; moderate, 2; strong, 3) and percentage score of immunoreactive tumor cells (<1%, 0; 1–25%, 1; 26–50%, 2; 51–75%, 3; >75%, 4).

The CD3 antibody (Abmart, #T55982, 1:100) and CD8 antibody (Abmart, #T40010, 1:100) were used to conduct immunohistochemical experiments related to immune infiltration, and the staining method was the same as above. The correlation between CD3, CD8 and CADM3 expression was detected by Wilcoxon rank sum test.

### CCK-8 assay

2 × 10^3^ cells were added to each hole of the 96-well plate and incubated at 37℃ for 24 h to make the cells stick to the wall. Then, 10µL CCK-8 reagent was added to each well for 2 h in the incubator. The absorbance of the cell at wavelength 450 nm was detected and the value was recorded.

### Colony formation assay

1 × 10^3^ cells were evenly spread on the six well plates and cultured at 37℃ until the cell colony was formed. The cells were fixed at about 30 min by 95% ethanol, followed by staining over 30 min with Crystal Violet Staining Solution. Clonal formation was counted after staining.

### Cell scratch assay

A marker or a steel needle was used to mark the outer side of the six-hole plate with two parallel lines spaced about 1.5 cm apart at the center of each well. Inoculated with 4 × 10^5^ cells at each well and cultured overnight. Then, a straight scratch was made in the middle of each hole in the six-hole plate perpendicular to the marked line. Each well was washed three times with sterile PBS to wash the cells scraped off in the previous step. The intersection of the scratch and the marking line was photographed and recorded as 0 h. Cells were further cultured under serum-free conditions. The above two steps were repeated after 12, 24 and 36 h, respectively. We measured each scratch edge line spacing with ImageJ after setting the scales and denoted as the scratch width. For each picture, the width was measured at three points and their average value was taken. The experimental group and control group were repeated three times by the above method, and the recorded data were quantitatively analyzed by multiple t-tests method. Cell mobility (%)=(0 h scratch width - scratch width after culture)/0 h scratch width 100%.

### Transwell assay

Transwell cells with a diameter of 8 μm were placed into a 24-well plate. 1 × 10^5^ cells were added into 150µL serum-free medium, and uniformly inoculated on the upper layer of the cells. Then 500µL complete medium was injected into the lower part of the chamber and cultured in the incubator for 6-12 h. The chambers were removed, the non-penetrating cells in the upper layer were erased. The cells were fixed by 4% paraformaldehyde for 30 min and stained by Crystal Violet for 30 min. The experimental group and control group were repeated three times by the above method. Using ImageJ to count cells, and the recorded data were quantitatively analyzed by t-test method.

### Data presentation and statistical analysis

SPSS (version 26.0) and R (version 4.2.1) were used for statistical analysis. 95% CI and HR were calculated using Cox univariate and multivariate models. We analyzed the correlation between clinical features and CADM3 expression using logistic regression. Set *P* < 0.05,| logFC| > 2 as the threshold to screen differential genes of CADM3. The correlation between CADM3 and immune infiltrated cells was examined by Pearson.

## Results

### CADM3 is low expressed in BC

**Fig. 1:**
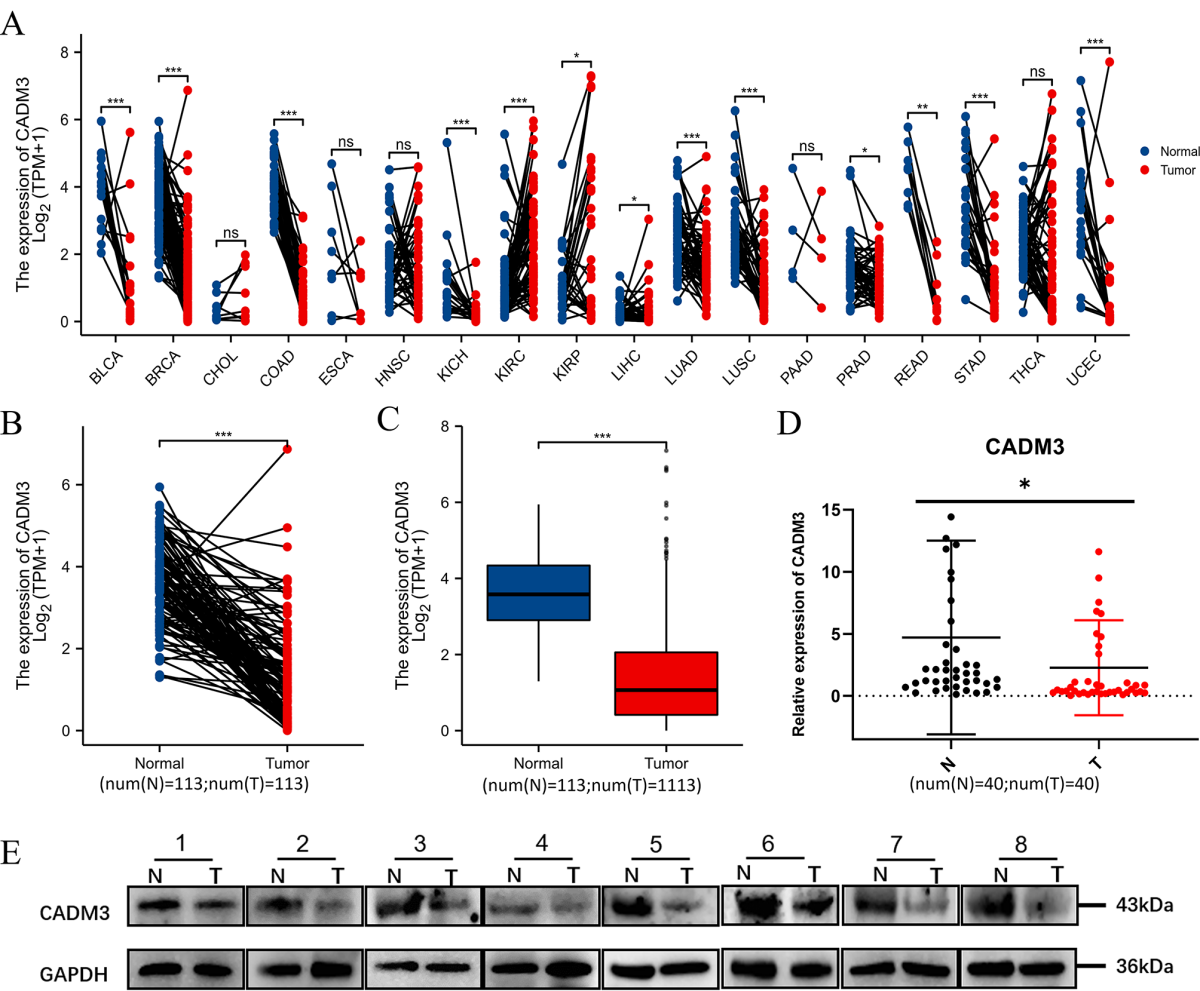
CADM3 was downregulated in human BC. **(A)** Pan-cancer analysis of CADM3 in TCGA database using Wilcoxon rank sum test. **(B)** Relative mRNA expression of CADM3 in the paired BC and normal tissues in TCGA database using Wilcoxon signed rank test. **(C)** Relative mRNA expression of CADM3 in the unpaired BC and normal tissues in TCGA database using Wilcoxon rank sum test. **(D)** mRNA expression level of CADM3 in 40 human BC tissues and their adjacent pairs of normal tissues. The statistical method selected was paired t test. **(E)** Protein expression of CADM3 in 8 cases of human BC and its adjacent pairs of normal tissues

Figure 1A showed pan-cancer analysis of CADM3 expression in 18 diverse human cancers. We found that CADM3 was significantly differentially expressed in 13 cancers, of which 11 were downregulated in cancer tissues, including BC. We further verified that CADM3 level was lower in BC tissues compared with normal breast tissues in TGCA database (Fig. 1B and C). Then, using fresh clinical tissue samples, we verified that the expression level of CADM3 in BC was lower than that of normal tissues from both protein and mRNA levels (Fig. 1D and E).

### High level of CADM3 infers a favorable prognosis in BC

**Fig. 2:**
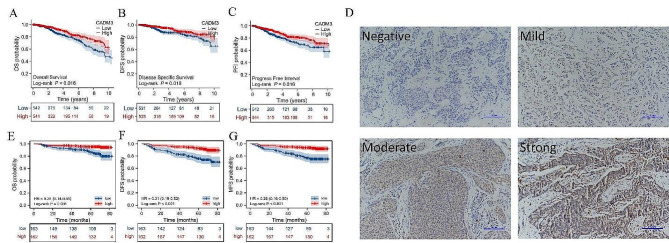
CADM3 upregulation was related to longer survival in patients with BC. The KM survival curve of BC patients were divided by CADM3 expression in TCGA database **(A-C)**: **(A)** OS, **(B)** DFS, **(C)** PFI. **(D)** CADM3 staining in paraffin sections of 325 human breast cancer tissues. The KM survival curve of breast cancer patients were plotted according to IHC staining results and patient follow-up information **(E-G)**: **(E)** OS, **(F)** DFS, **(G)** MFS. The log-rank test was used for the above survival analysis

We first draw the KM survival curve through the TCGA database. We divided the patients into two groups by the median expression of CADM3. Overall survival (OS) analysis, disease free survival (DFS) analysis and progression free interval (PFI) analysis all indicated that, high level of CADM3 infered a favorable prognosis in BC with statistically significant differences (Fig. 2A-C).

We then performed immunohistochemical staining of paraffin sections from 325 BC patients operated on in 2015 (Fig. 2D). Patients were classified into high expression and low expression groups by staining intensity and distribution scores. According to the patient Kaplan Meier survival analysis based on CADM3 expression, BC patients with low CADM3 level had shorter OS, DFS and metastasis-free survival (MFS) (Fig. 2E-G), which was consistent with the results of the TCGA database prediction.

**Fig. 3:**
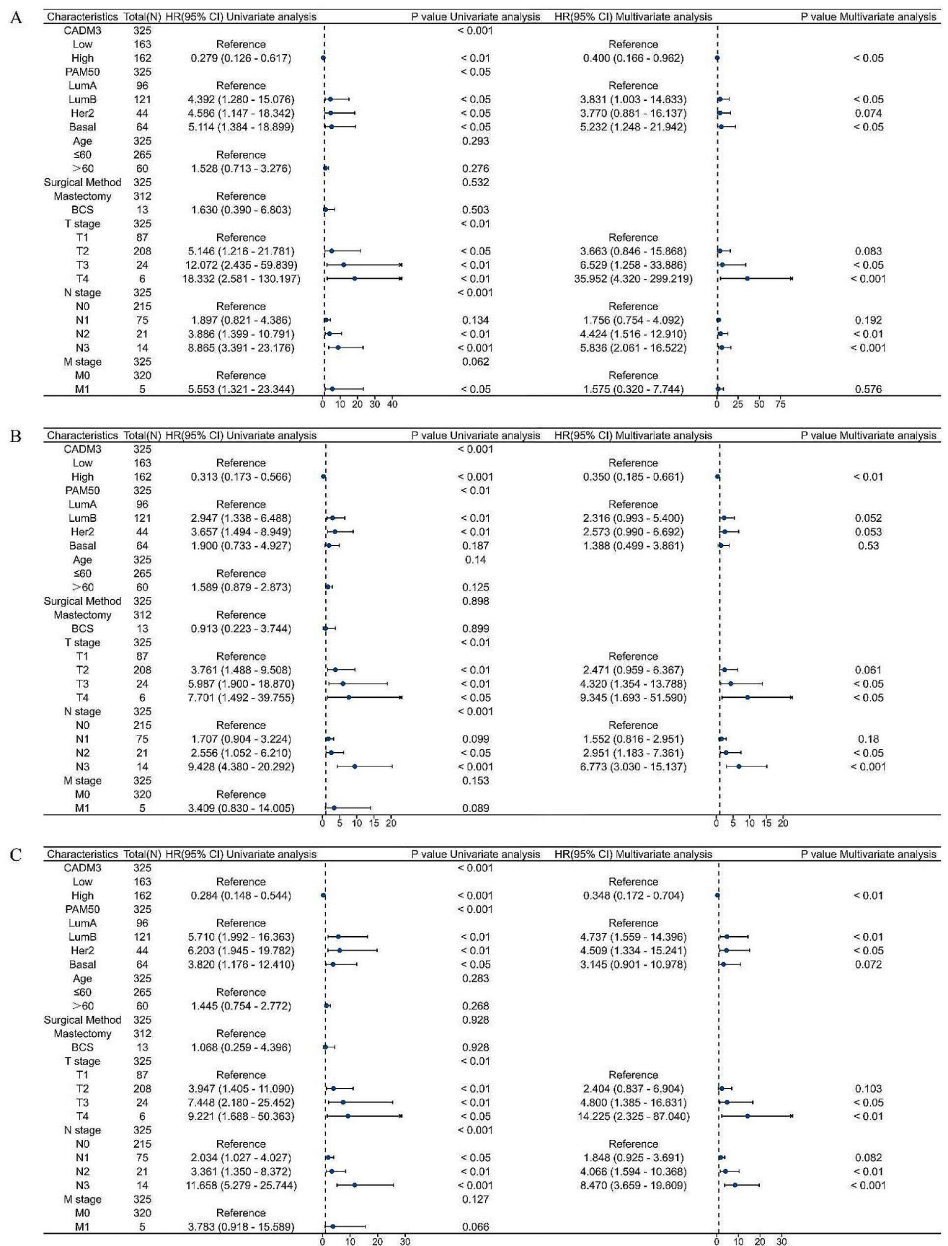
The forest map of the cox regression analysis of the relationship between CADM3 expression and OS, DFS, MFS of BC patients **(A-C)**: **(A)** OS, **(B)** DFS, **(C)** MFS

On this basis, cox regression analysis was used to conduct in-depth exploration of clinical BC sample follow-up data to determine the correlation between CADM3 and other clinical multivariate features and survival of BC patients. According to the univariate analysis, a number of factors including CADM3 expression, molecular typing (PAM50), tumor status (T), and lymph node status (N) were notably related to OS, DFS, and MFS (Fig. 3A-C). Multivariate analysis indicated that CADM3 level was an independent prognostic factor of BC. Table 1 presents patient baseline data. In particular, these 325 patients were collected from our clinical collection and did not use a public database.

**Table 1:** Baseline of 325 patients with breast cancer

	Total	CADM3 LOW	CADM3 High	p-value
**All patients**	325	163	162	
**Age**				<0.001
**≤ 60**	265(81.5)	120(73.6)	145(89.5)	
**>60**	60(18.5)	43(26.4)	17(10.5)	
**PAM50**				<0.001
**LumA**	96(29.5)	28(17.2)	68(42.0)^*^	
**LumB**	121(37.2)	62(38.0)	59(36.4)	
**Her2**	44(13.5)	23(14.1)	21(13.0)	
**Basal**	64(19.7)	50(30.7)	14(8.6)^*^	
**Surgical Method**				0.402
**Mastectomy**	312(96.0)	155(95.1)	157(96.9)	
**BCS**	13(4.0)	8(4.9)	5(3.1)	
**T stage**				0.046
**Tis**	9(2.8)	3(1.8)	6(3.7)	
**T1**	78(24.0)	31(19.0)	47(29.0)^*^	
**T2**	208(64.0)	116(71.2)	92(56.8)^*^	
**T3**	24(7.4)	12(7.4)	12(7.4)	
**T4**	6(1.8)	1(0.6)	5(3.1)	
**N stage**				0.951
**N0**	215(66.2)	107(65.6)	108(66.7)	
**N1**	75(23.1)	37(22.7)	38(23.5)	
**N2**	21(6.5)	11(6.7)	10(6.2)	
**N3**	14(4.3)	8(4.9)	6(3.7)	
**M stage**				0.179
**M0**	320(98.5)	159(97.5)	161(99.4)	
**M1**	5(1.5)	4(2.5)	1(0.6)	

* indicates a statistical difference compared with the group with low CADM3 expression.

### CADM3 expression correlates with clinical characteristics in BC

**Fig. 4:**
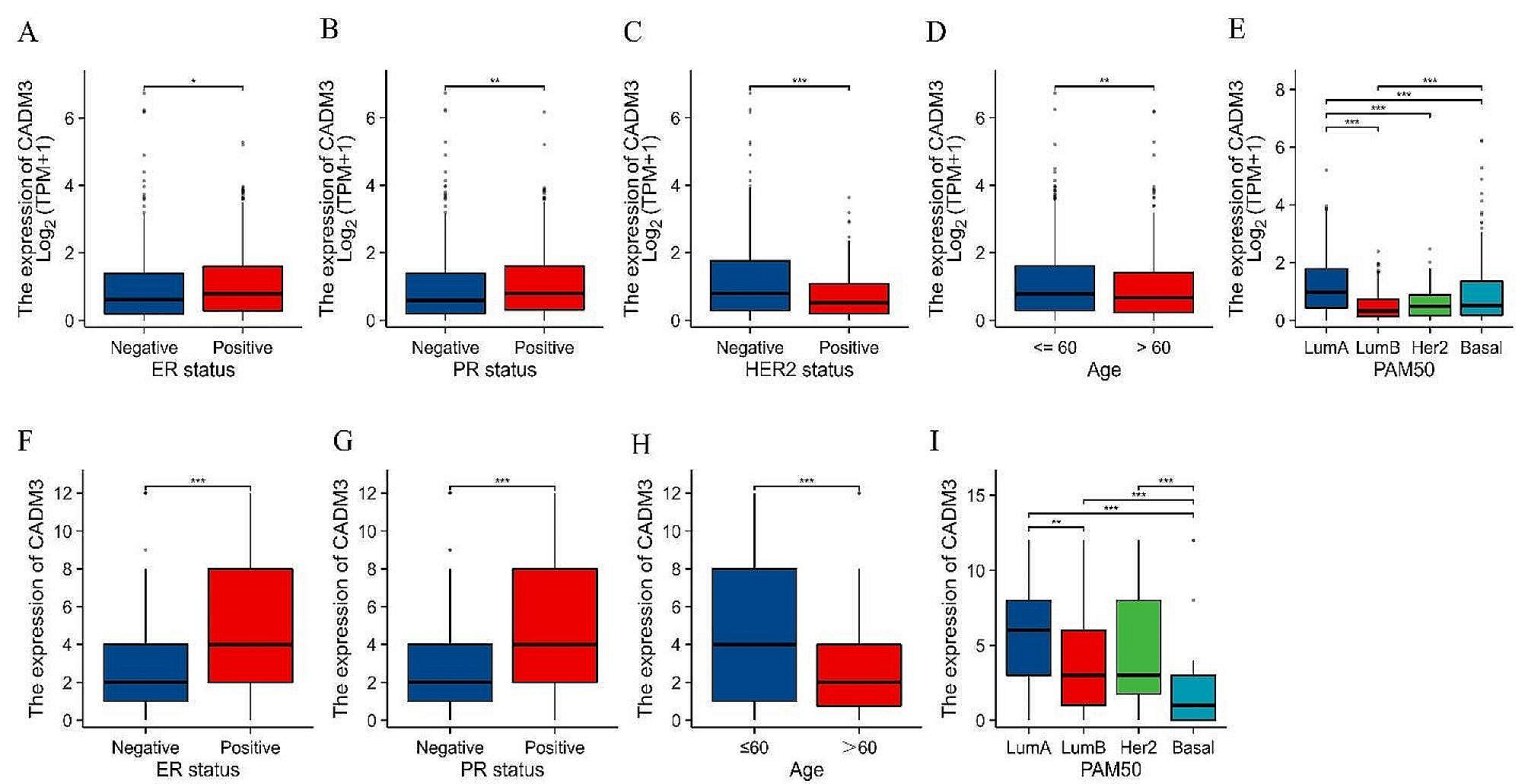
The relationship between CADM3 and the clinical characteristics of BC patients in TCGA **(A-E)**: **(A)** ER status, **(B)** PR status, **(C)** HER-2 status, **(D)** age, **(E)** PAM50 subtypes. The association between CADM3 and clinical features in clinical samples **(F-I)**: **(F)** ER status, **(G)** PR status, **(H)** age, **(I)** PAM50 subtypes. Wilcoxon rank sum test was used for statistical analysis of ER, PR, HER2 status and age group. Kruskal-Wallis Test and Dunn’s test were used for the PAM50 group

We evaluated the correlation between CADM3 expression level and various clinical characteristics and pathological parameters using TCGA database. As a result, the increased CADM3 level in BC was considerably related to ER (*P* < 0.05; Fig. 4A), PR (*P* < 0.01; Fig. 4B), HER2 (*P* < 0.001; Fig. 4C) expression status, age (*P* < 0.01; Fig. 4D) and PAM50 (*P* < 0.001; Fig. 4E). These results indicated that BC patients with high CADM3 are more likely to be ER and PR positive, HER2 negative, age ≤ 60 years, and luminal A subtype than those with low CADM3 (Table 2).

**Table 2:** Association between CADM3 expression and clinicopathological features from the TCGA database

Comparison	Statistical significance
ER status	
Negative -vs- Positive	0.0351
PR status	
Negative -vs- Positive	0.0011
HER2 status	
Negative -vs- Positive	0.0003
Age	
≤ 60 -vs->60	0.0093
PAM50	
LumA -vs- LumB	8.24e-24
LumA -vs- Her2	8.57e-08
LumA -vs- Basal	8.6e-07
LumB -vs- Her2	1.0000
LumB -vs- Basal	0.0004
Her2 -vs- Basal	0.4596

Next, combining immunohistochemical staining results and clinical patient data, we further evaluated the connection between CADM3 and various clinical features and pathological parameters in 325 BC patients. The results indicated that the increase in CADM3 was connected to ER (*P* < 0.001; Fig. 4F), PR status (*P* < 0.001; Fig. 4G), age (*P* < 0.001; Fig. 4H) and PAM50 (*P* < 0.001; Fig. 4I). These results agreed well with the analysis in TCGA database, indicating that BC patients with high CADM3 tend to have ER, PR positive, age ≤ 60 years, and luminal A subtype (Table 3).

**Table 3:** Association between CADM3 expression and clinicopathological features in 325 clinical samples

Comparison	Statistical significance
ER status	
Negative -vs- Positive	3.91e-07
PR status	
Negative -vs- Positive	1.35e-05
Age	
≤ 60 -vs->60	0.0002
PAM50	
LumA -vs- LumB	0.0062
LumA -vs- Her2	0.0929
LumA -vs- Basal	5.39e-14
LumB -vs- Her2	1.0000
LumB -vs- Basal	1.24e-06
Her2 -vs- Basal	0.0002

### High expression of CADM3 suppressed proliferation and migration of BC cells

**Fig. 5:**
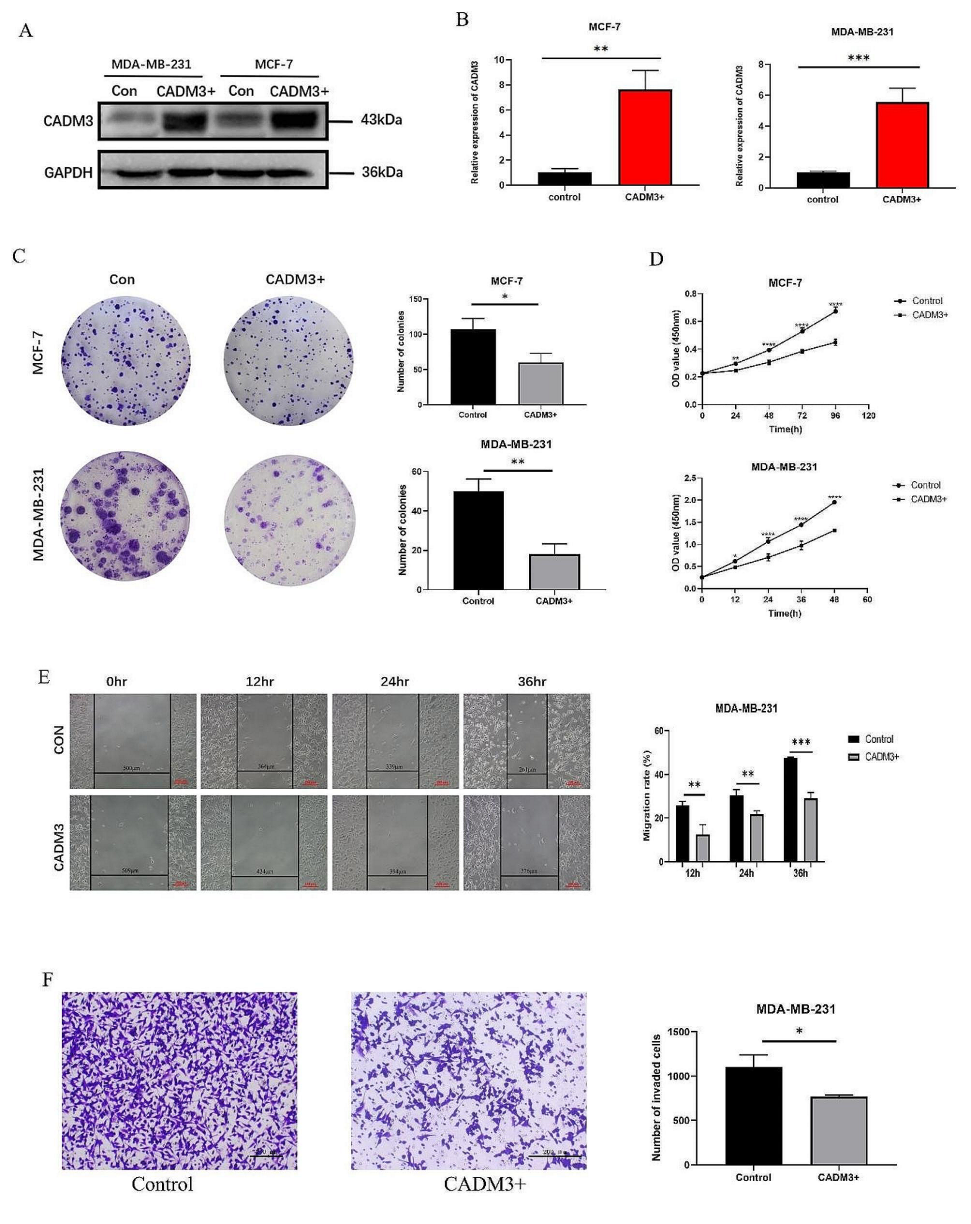
The overexpression efficiency of CADM3 in MCF-7 and MDA-MB-231 cell lines verified by **(A)** WB and **(B)** qPCR. **(C)** Results of clonal formation assay. The cell colonies of the three groups were counted, and the data were presented as the mean values of three independent experiments (*p < 0.05, **p < 0.01, *n* = 3). **(D)** Results of CCK-8 experiment. CCK-8 was added to MCF-7 cells at 0, 24, 48, 72, and 96 h of cell adherence, while MDA-MB-231 cells were added to CCK-8 at 0, 12, 24, 36, and 48 h of cell adherence. Data showed the mean of three independent experiments (*p < 0.05, **p < 0.01, ****p < 0.0001, *n* = 3). **(E)** Results of cell scratch assay. Results of cell scratch assay. The scratch widths of the experimental group and the control group at 0, 12, 24 and 36 h were observed respectively, and the data were presented as the mean values of three independent experiments (**p < 0.01, ***p < 0.001, *n* = 3). **(F)** Results of transwell assay. Quantization map data showed the mean values of three independent experiments (*p < 0.05, *n* = 3)

To determine whether high level of CADM3 contribute to the inhibition of BC cell proliferation, we constructed stable overexpression cell lines of CADM3 using MCF-7 and MDA-MB-231 cell lines. We verified the construction results from the protein level and mRNA level respectively, and we found that the relative expression level of CADM3 in the overexpression group was much higher than the control group (Fig. 5A and B).

For the purpose of exploring the effect of CADM3 on proliferation of BC, we conducted clonal formation assay and CCK-8 assay in CADM3 overexpression group and the control group, using MCF-7 and MDA-MB-231 cell lines. Cloning experiments confirmed that the number of colonies formed by CADM3 overexpressed cells was notably lower than that of the control cells (Fig. 5C). Similarly, in the CCK-8 experiment, the proliferation rate of CADM3 overexpressed cells was clearly slower than the control group (Fig. 5D).

To investigate the effect of CADM3 on migration of BC, we conducted cell scratch assay and transwell assay on MDA-MB-231 cell line. It was proven that the scratch healing rate of CADM3 overexpressed group was lower than the control group (Fig. 5E). According to the transwell experiment, fewer cells passed through the chamber in the CADM3 overexpressed group than the control group (Fig. 5F).

### CADM3 and immune infiltration

**Fig. 6:**
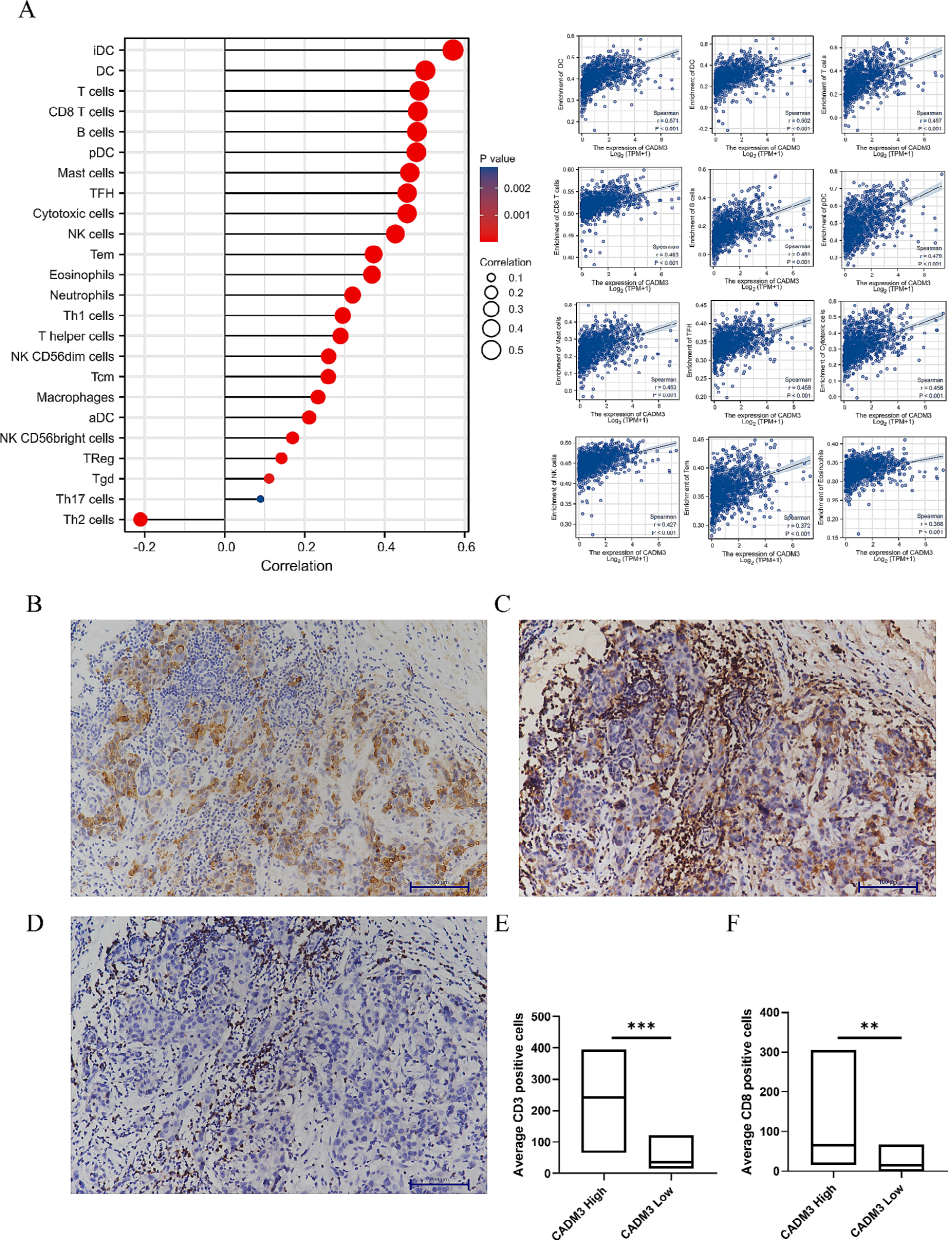
**(A)** Correlation between CADM3 expression and multiple kinds of tumor infiltrating immune cells: iDC cells, DC cells, T cells, CD8 T cells, B cells, pDC cells, mast cells, TFH cells, cytotoxic cells, NK cells, Tem cells, eosinophils cells. **(B-D)** CADM3 expression was associated with tumor-infiltrating immune cells in BC patients: Representative immunohistochemical staining of CADM3 **(B)**, CD3 **(C)** and CD8 **(D)** in one patient with BC (×200). **(E-F)** Correlation between CADM3 expression and the numbers of CD3 and CD8 positive cells manually counted under × 200 magnification in BC samples (**p < 0.01, ***p < 0.001). The statistical analysis was wilcoxon rank sum test

Through comprehensive analysis of the TCGA database, we verified that CADM3 expression in BC was closely related to immune infiltrating cells. The results showed that the expression of CADM3 was positively associated with infiltration levels of iDC cells, dendritic cells (DC), T cells, CD8 T cells, B cells, pDC cells, mast cells, T follicle helper cells (TFH), cytotoxic T cells, NK cells, T effector memory cells (Tem), and eosinophils (Fig. 6A).

### Function prediction of CADM3-related genes

**Fig. 7:**
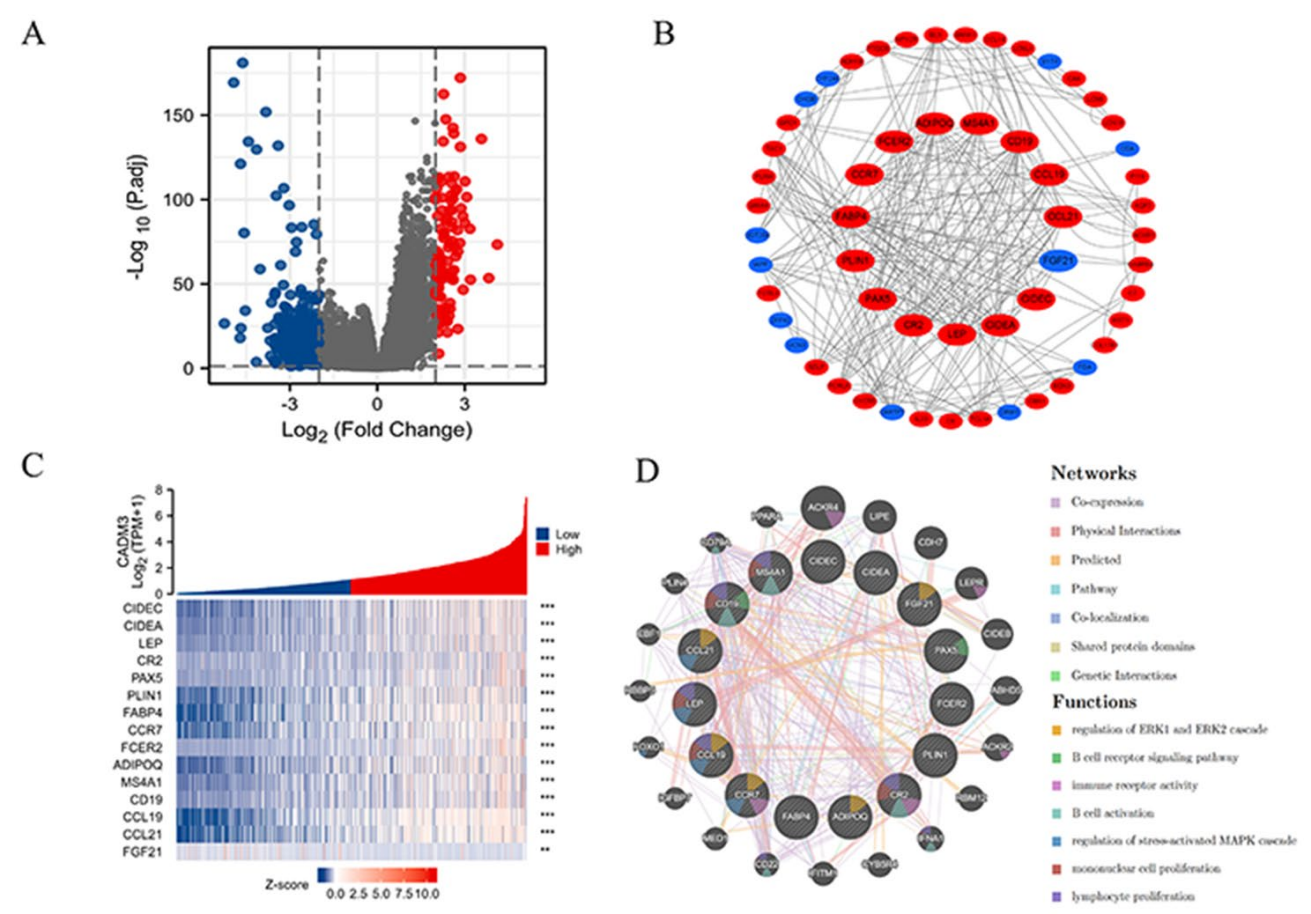
**(A)** Volcano maps of DEGs from TCGA. **(B)** PPI network of the 15 hub genes made by STRING and cytoscape. **(C)** Co-expression heatmap of the 15 hub genes. The statistical method was Spearman. **(D)** PPI network and functional analysis of the 15 hub genes. The inner circle represented the input genes and the outer circle corresponded to GeneMANIA proposed hub genes. Size of the circles suggested the association with the input genes. The statistical analysis used for the above was Spearman

We performed CADM3 single gene differential expression analysis on invasive BC data in the TCGA database, and visualized the results using volcano maps (Fig. 7A). According to the condition of *P* < 0.05 and| log FC| > 2, we selected 711 of the genes, and we got the 15 hub genes using MCC algorithm in cytoscape. Among them, 14 genes showed positive correlation with CADM3 and one showed negative correlation with CADM3. Protein interaction networks of 15 hub genes (Fig. 7B) and their co-expression heat maps (Fig. 7C) were made. In addition, we found the functions of those genes were mainly related to the regulation of ERK1 and ERK2 cascade, B cell receptor signaling pathway, immune receptor activity, B cell activation, regulation of stress-activated MAPK cascade, mononuclear cell proliferation and lymphocyte proliferation (Fig. 7D).

### Correlation between CADM3 and MAPK pathway

**Fig. 8:**
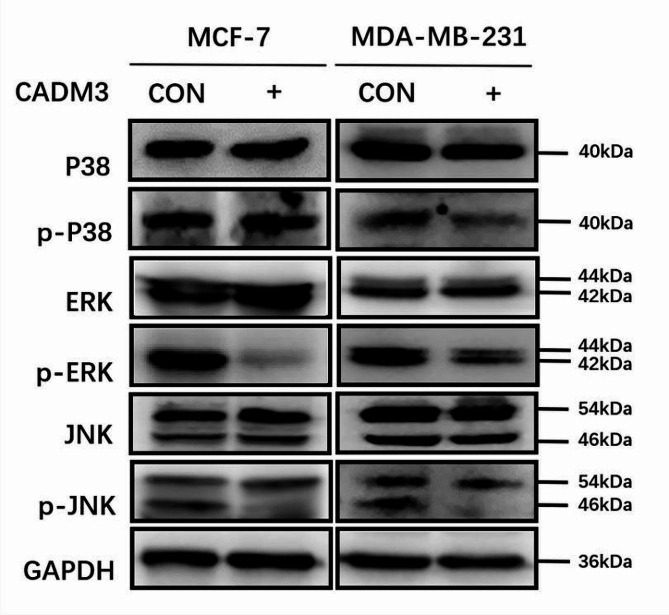
The expressions of key proteins P38, ERK, JNK and their phosphorylated proteins in MAPK pathway were verified by WB assay

The result of gene function prediction showed that the effect of CADM3 on the biological behavior of BC cells may be associated with MAPK pathway. We verified the expression of the key proteins of MAPK pathway: P38, ERK, JNK, and the expression levels of their phosphorylated proteins. The results showed that ERK1/ERK2 protein phosphorylation and JNK1 protein phosphorylation were inhibited in MCF-7 and MDA-MB-231 cell lines with high level of CADM3. There was no considerable difference in phosphorylation of P38 protein (Fig. 8).

## Discussion

In our research, the TCGA database was used to explore the CADM3 expression level in BC tissues and adjacent normal tissues, and the correlation between CADM3 expression level and ER, PR, HER2 status, age and PAM50, as well as the association between CADM3 and patient prognosis. Immunohistochemical staining, WB and qPCR results indicated that CADM3 was low expressed in BC, and highly expressed in ER +, PR +, age ≤ 60 and luminal A type BC patients. Analysis of the TCGA database and clinical sample data showed that highly expression of CADM3 is related to longer OS, DFS and MFS, and CADM3 can be used as an independent prognostic factor in BC.

BC is highly heterogeneous, with great individual differences among different patients [22]. At the same time, the occurrence and development of BC are also very complex, including various biological processes, such as tumor proliferation, invasion and angiogenesis, as well as significant possibility of metastasis and recurrence [23–26]. 90% of BC recurrence is local recurrence, which is caused by residual cancer cells [27]. Studies have shown that residual cancer cells will persist, resulting in recurrence of the tumor and failure in treatment [28]. Therefore, it is very important to explore the key regulatory factors of BC cell proliferation, invasion and migration to prevent cancer recurrence. Cell adhesion factors (CADMs) play an crucial part in occurrence, invasion and migration of tumors [11, 29]. Thereinto, CADM1 has been comprehensively studied, and its inhibitory effect on tumor proliferation and invasion has been reported in ovarian cancer [6], bladder cancer [10] and other tumors. Our research found that CADM3 suppressed the proliferation of BC cells through colony formation experiment and CCK-8 experiment. Transwell assay and cell scratch assay found that CADM3 inhibited the migration of BC cells. These results indicated that CADM3 is of great importance in delaying the progression of BC by suppressing proliferation and migration of BC cells, and then inhibiting the growth and metastasis of BC.

Mitogen-activated protein kinases (MAPK) are a class of serine/threonine protein kinases broadly exist in cytoplasm, and enter the nucleus after activation to activate target genes [30]. MAPK pathway mainly includes p38, ERK and JNK pathways. p38 pathway participates in proliferation, differentiation and apoptosis. After phosphorylation, p38 enters the nucleus and affects biological processes such as protein synthesis and gene transcription by regulating the transcription factors [31]. JNK may not only regulate various transcription factors, including c-Jun, c-Fos, ATF-2, AP-1, p53 and Elk, but phosphorylate a variety of cytoplasmic substrates, such as cytoskeletal proteins and mitochondrial proteins after activation, and eventually leading to cell apoptosis [32, 33]. JNK and p38 are more related to response to environmental and endogenous stress signals, but their differences are not yet clear and there are several overlapping between functions [34, 35]. The ERK pathway is also one of the MAPK signaling pathways. The extracellular signals that control key cellular processes were transmitted to cytoplasmic and nuclear effectors by the ERK cascade. It is a signal transduction module for vital cellular processes, such as proliferation, migration and apoptosis. Dysregulation of the cascade is often associated with the development of cancer [34, 36]. Compared with normal tissues, ERK may be activated or overexpressed in BC [37, 38], but the conclusion about the relevance of phosphorylation of ERK1/2 to the prognosis of BC is still controversial. Researches have emphasized the correlation between the expression of nuclear phosphorylation of ERK1/2 and good prognosis characteristics [39, 40]. Other studies have found that nuclear phosphorylation of ERK1/2 has association with poor prognosis of BC [41, 42]. The correlation between phosphorylation and prognosis of BC needs to be further explored.

At present, there are few studies on CADMs and MAPK pathway. Cai et al. [43] found in 2018 that CADM1 is involved in the regulation of tumor formation by participating in MAPK signaling pathway. In this study, we predicted that the effects of CADM3 on the biological behavior of BC cells might have association with MAPK pathway through gene function prediction. WB assay results showed that ERK1/2 and JNK1 protein phosphorylation were inhibited in MCF-7 and MD-MB-231 cell lines with high level of CADM3. These results suggested that CADM3 may affect proliferation, migration and other cell biological behaviors of BC by inhibiting MAPK pathway protein phosphorylation, and its specific mechanism remains to be further studied.

It is well known that tumor immune infiltration can affect patients’ sensitivity to chemotherapy, radiotherapy and immunotherapy, as well as the survival of cancer patients [44–46]. The beneficial role of T cells including CD8 T cells [47, 48] and some subtypes of CD4 T cells such as TFH ^4^ [49] in BC has been revealed. DCs acts as a tumor antigen transporter to initiate T cell activation, which is required for T-cell-dependent immunity and ICI therapeutic responses [50, 51]. NK cells can exert cytotoxic effects in BC, thereby increasing the levels of death receptors [52, 53]. In addition, the antitumor role of B cells [54, 55], eosinophils [56] in BC has also been demonstrated by strong evidence. Previous studies have shown that there is a correlation between the CADMs family and immune infiltrating cells, and its silencing may be associated with tumor immune evasion mechanisms [11–13]. In this study, through TCGA database analysis, we found that CADM3 was closely related to the above immune infiltrating cells, suggesting that the high expression of CADM3 may improve the prognosis of BC by promoting anti-tumor immunity.

ERK pathway plays an important role in immunotherapy. The activation of ERK pathway can drive the apparent activation of super enhancer PD-L1L2-SE, the homeoregulatory element of PD-L1, and then up-regulate the expression of PD-L1 in tumor cells [57, 58]. The up-regulation of PD-L1 expression in tumor cells will further lead to the inactivation of T cells and cause immunosuppression. Blocking ERK pathway can significantly enhance the efficacy of immunotherapy [59]. Our study showed that upregulation of CADM3 could inhibit ERK protein phosphorylation in the MAPK pathway, thereby inhibiting ERK pathway activation. At the same time, we found that the up-regulation of CADM3 was positively correlated with the expression of T cell markers CD3 and CD8. Therefore, we speculate that CADM3 inhibits PD-L1 expression in breast cancer cells by regulating ERK protein in the MAPK pathway, thereby reducing its binding to T cells, so that T cells are released and the weakened immune function of tumor cells is restored.

However, there are still some limitations in this study: we did not construct a stable cell line with low CADM3 knockdown, resulting in insufficient research. We have not verified the expression of CADM3 and its regulatory effect on MAPK pathway in clinical specimens, and have not yet explored the specific mechanism of its role. Lack of experimental verification of immune correlation. In the follow-up work, we may further improve.

In conclusion, CADM3 plays an inhibitory part in the progression of BC, which has not been studied yet. With the further exploration of its function and mechanism, CADM3 may become a new therapeutic target for BC.

### Electronic supplementary material

Below is the link to the electronic supplementary material.

Supplementary Material 1

## Data Availability

The datasets used and/or analysed during the current study available from the corresponding author on reasonable request.

## Abbreviations

BC: breast cancer
CADM3: cell adhesion molecule 3
TCGA: The Cancer Genome Altas
PPI: protein-protein interaction
IHC: immunohistochemical
GSEA: gene set enrichment analysis
TPM: transcripts per million reads
WB: western blot
KM: Kaplan-Meier
DEGs: differentially expressed genes
GSVA: Gene Set Variation Analysis
OS: overall survival
DFS: disease specific survival
PFI: progress free interval
RFS: recurrence-free survival
MFS: metastasis-free survival
HR: hazard ratio
CI: confidence interval
MAPK: mitogen activated protein kinase
DCs: dendritic cells
Tem: T effector memory cell

## References

[1] SUNG H, FERLAY J, SIEGEL RL (2021). Global Cancer statistics 2020: GLOBOCAN estimates of incidence and Mortality Worldwide for 36 cancers in 185 countries [J]. Cancer J Clin.

[2] WAKS A G, WINER EP (2019). Breast Cancer Treatment: a review [J]. JAMA.

[3] ELANGBAM C S, QUALLS C W JR (1997). DAHLGREN R R. Cell adhesion molecules–update [J]. Vet Pathol.

[4] EDELMAN G M (1986). Cell adhesion molecules in the regulation of animal form and tissue pattern [J]. Annu Rev Cell Biol.

[5] TAKAI Y (2003). Nectin and Afadin: novel organizers of intercellular junctions [J]. J Cell Sci.

[6] SI X, XU F, XU F, et al. CADM1 inhibits ovarian cancer cell proliferation and migration by potentially regulating the PI3K/Akt/mTOR pathway [J]. Volume 123. Biomedicine & pharmacotherapy = Biomedecine & pharmacotherapie; 2020. p. 109717. 10.1016/j.biopha.2019.109717 31865146

[7] BOTLING J, EDLUND K, LOHR M (2013). Biomarker discovery in non-small cell lung cancer: integrating gene expression profiling, meta-analysis, and tissue microarray validation [J]. Clin cancer Research: Official J Am Association Cancer Res.

[8] CHANG H, MA M, MA R (2016). Folate deficiency and aberrant expression of cell adhesion molecule 1 are potential indicators of prognosis in laryngeal squamous cell carcinoma [J]. Oncol Lett.

[9] CAI Q, ZHU A. GONG L. Exosomes of glioma cells deliver miR-148a to promote proliferation and metastasis of glioblastoma via targeting CADM1 [J]. Bulletin du cancer, 2018, 105(7–8): 643– 51. 10.1016/j.bulcan.2018.05.003 29921422

[10] CHEN Y, LIU L, GUO Z (2019). Lost expression of cell adhesion molecule 1 is associated with bladder cancer progression and recurrence and its overexpression inhibited tumor cell malignant behaviors [J]. Oncol Lett.

[11] HIROHASHI S, KANAI Y (2003). Cell adhesion system and human cancer morphogenesis [J]. Cancer Sci.

[12] BOLES KS, BARCHET W, DIACOVO T (2005). The tumor suppressor TSLC1/NECL-2 triggers NK-cell and CD8 + T-cell responses through the cell-surface receptor CRTAM [J]. Blood.

[13] OBERNDORFER F, MüLLAUER L (2018). Molecular pathology of lung cancer: current status and perspectives [J]. Curr Opin Oncol.

[14] FUJITO T, IKEDA W (2005). Inhibition of cell movement and proliferation by cell-cell contact-induced interaction of Necl-5 with nectin-3 [J]. J Cell Biol.

[15] LOVE M I, HUBER W (2014). Moderated estimation of Fold change and dispersion for RNA-seq data with DESeq2 [J]. Genome Biol.

[16] ROBINSON MD, MCCARTHY D J, SMYTH, G K. Bioinf (Oxford England). 2010;26(1):139–40. edgeR: a Bioconductor package for differential expression analysis of digital gene expression data [J]. 10.1093/bioinformatics/btp616 PMC2796818 19910308

[17] HäNZELMANN S, CASTELO R. BMC Bioinformatics. 2013;14:7. GUINNEY J. GSVA: gene set variation analysis for microarray and RNA-seq data [J]. 10.1186/1471-2105-14-7 PMC3618321 23323831

[18] BINDEA G, MLECNIK B (2013). Spatiotemporal dynamics of intratumoral immune cells reveal the immune landscape in human cancer [J]. Immunity.

[19] SZKLARCZYK D, GABLE A L, LYON D (2019). STRING v11: protein-protein association networks with increased coverage, supporting functional discovery in genome-wide experimental datasets [J]. Nucleic Acids Res.

[20] CHIN C H, CHEN S H, WU H H (2014). cytoHubba: identifying hub objects and sub-networks from complex interactome [J]. BMC Syst Biol.

[21] WARDE-FARLEY D, DONALDSON S L, COMES O et al. The GeneMANIA prediction server: biological network integration for gene prioritization and predicting gene function [J]. Nucleic Acids Res, 2010, 38(Web Server issue): W214–20. 10.1093/nar/gkq537 PMC2896186 20576703

[22] POLYAK K (2011). Heterogeneity in breast cancer [J]. J Clin Investig.

[23] CASTAñEDA-GILL JM, VISHWANATHA JK (2016). Antiangiogenic mechanisms and factors in breast cancer treatment [J]. J Carcinog.

[24] KARAGIANNIS GS, JONES J G GOSWAMIS (2016). Signatures of breast cancer metastasis at a glance [J]. J Cell Sci.

[25] POLYAK K (2007). Breast cancer: origins and evolution [J]. J Clin Investig.

[26] AHMAD A. Pathways to breast cancer recurrence [J]. ISRN oncology. 2013, 2013: 290568. 10.1155/2013/290568 PMC3603357 23533807

[27] LU H, GUO Y, GUPTA G (2019). Mitogen-activated protein kinase (MAPK): new insights in breast Cancer [J]. J Environ Pathol Toxicol Oncology: Official Organ Int Soc Environ Toxicol Cancer.

[28] BLATTER S, ROTTENBERG S. Minimal residual disease in cancer therapy–small things make all the difference [J]. Drug resistance updates: reviews and commentaries in antimicrobial and anticancer chemotherapy, 2015, 21–2: 1–10. 10.1016/j.drup.2015.08.003 26307504

[29] MURAKAMI Y (2005). Involvement of a cell adhesion molecule, TSLC1/IGSF4, in human oncogenesis [J]. Cancer Sci.

[30] XU C, LIU R, ZHANG Q (2017). The diversification of evolutionarily conserved MAPK cascades correlates with the evolution of Fungal species and Development of lifestyles [J]. Genome Biol Evol.

[31] ZHANG Y, SMOLEN P, BAXTER D A et al. Biphasic regulation of p38 MAPK by Serotonin contributes to the efficacy of stimulus protocols that induce long-term synaptic facilitation [J]. eNeuro, 2017, 4(1). 10.1523/ENEURO.0373-16.2017 PMC5307297 28197555

[32] JOHNSON GL (2007). The c-jun kinase/stress-activated pathway: regulation, function and role in human disease [J]. Biochim Biophys Acta.

[33] CUI J, ZHANG M, ZHANG Y Q (2007). JNK pathway: diseases and therapeutic potential [J]. Acta Pharmacol Sin.

[34] DHILLON A S, HAGAN S, RATH O (2007). MAP kinase signalling pathways in cancer [J]. Oncogene.

[35] LEE S, RAUCH J. KOLCH W. Targeting MAPK signaling in Cancer: mechanisms of Drug Resistance and sensitivity [J]. Int J Mol Sci, 2020, 21(3). 10.3390/ijms21031102 PMC7037308 32046099

[36] AVRUCH J (2007). MAP kinase pathways: the first twenty years [J]. Biochim Biophys Acta.

[37] SIVARAMAN VS, WANG H, NUOVO G J (1997). Hyperexpression of mitogen-activated protein kinase in human breast cancer [J]. J Clin Investig.

[38] MUELLER H, FLURY N, EPPENBERGER-CASTORI S (2000). Potential prognostic value of mitogen-activated protein kinase activity for disease-free survival of primary breast cancer patients [J]. Int J Cancer.

[39] JERJEES D A, ALABDULLAH M, ALKAABI M (2014). ERK1/2 is related to oestrogen receptor and predicts outcome in hormone-treated breast cancer [J]. Breast Cancer Res Treat.

[40] MILDE-LANGOSCH K, BAMBERGER A M, RIECK G (2005). Expression and prognostic relevance of activated extracellular-regulated kinases (ERK1/2) in breast cancer [J]. Br J Cancer.

[41] KUO H T, HSU H T, CHANG C C (2013). High nuclear phosphorylated extracellular signal-regulated kinase expression associated with poor differentiation, larger tumor size, and an advanced stage of breast cancer [J]. Pol J Pathology: Official J Pol Soc Pathologists.

[42] NAKOPOULOU L, MYLONA E, RAFAILIDIS P et al. Effect of different ERK2 protein localizations on prognosis of patients with invasive breast carcinoma [J]. APMIS: acta pathologica, microbiologica, et immunologica Scandinavica, 2005, 113(10): 693–701. 10.1111/j.1600-0463.2005.apm_236.x 16309429

[43] CAI H, MIAO M (2018). Mir-214-3p promotes the proliferation, migration and invasion of osteosarcoma cells by targeting CADM1 [J]. Oncol Lett.

[44] WANICZEK D, LORENC Z, ŚNIETURA M et al. Tumor-Associated Macrophages and Regulatory T Cells Infiltration and the clinical outcome in Colorectal Cancer [J]. Archivum Immunologiae et therapiae experimentalis, 2017, 65(5): 445–54. 10.1007/s00005-017-0463-9 PMC5602054 28343267

[45] LYU L, YAO J, WANG M (2020). Overexpressed pseudogene HLA-DPB2 promotes Tumor Immune infiltrates by regulating HLA-DPB1 and indicates a better prognosis in breast Cancer [J]. Front Oncol.

[46] YE L, ZHANG T, KANG Z (2019). Tumor-infiltrating Immune cells Act as a marker for prognosis in Colorectal Cancer [J]. Front Immunol.

[47] MAHMOUD S M, PAISH E C, POWE D G (2011). Tumor-infiltrating CD8 + lymphocytes predict clinical outcome in breast cancer [J]. J Clin Oncology: Official J Am Soc Clin Oncol.

[48] ALI H, R, PROVENZANO E, DAWSON S J (2014). Association between CD8 + T-cell infiltration and breast cancer survival in 12,439 patients [J]. Annals Oncology: Official J Eur Soc Med Oncol.

[49] GU-TRANTIEN C, LOI S (2013). CD4^+^ follicular helper T cell infiltration predicts breast cancer survival [J]. J Clin Investig.

[50] BROZ ML, BINNEWIES M (2014). Dissecting the tumor myeloid compartment reveals rare activating antigen-presenting cells critical for T cell immunity [J]. Cancer Cell.

[51] SáNCHEZ-PAULETE A R, CUETO F J, MARTíNEZ-LóPEZ M (2016). Cancer Immunotherapy with Immunomodulatory Anti-CD137 and Anti-PD-1 monoclonal antibodies requires BATF3-Dependent dendritic cells [J]. Cancer Discov.

[52] SCHAAF M B, GARG A D AGOSTINISP. Defining the role of the tumor vasculature in antitumor immunity and immunotherapy [J]. Volume 9. Cell death & disease; 2018. p. 115. 2. 10.1038/s41419-017-0061-0 PMC5833710 29371595

[53] KIM H W, KIM J E, HWANG M H (2013). Enhancement of natural killer cell cytotoxicity by sodium/iodide symporter gene-mediated radioiodine pretreatment in breast cancer cells [J]. PLoS ONE.

[54] NELSON B H (2010). CD20 + B cells: the other tumor-infiltrating lymphocytes [J]. J Immunol (Baltimore Md: 1950).

[55] IGLESIA MD, VINCENT B G, PARKER JS (2014). Prognostic B-cell signatures using mRNA-seq in patients with subtype-specific breast and ovarian cancer [J]. Clin cancer Research: Official J Am Association Cancer Res.

[56] PONCIN A, ONESTI C E, JOSSE C et al. Immunity and breast Cancer: focus on eosinophils [J]. Biomedicines, 2021, 9(9). 10.3390/biomedicines9091087 PMC8470317 34572273

[57] XU Y, WU Y, ZHANG S (2020). A tumor-specific Super-enhancer drives Immune Evasion by Guiding Synchronous expression of PD-L1 and PD-L2 [J]. Cell Rep.

[58] MA P, JIN X, FAN Z (2021). Super-enhancer receives signals from the extracellular matrix to induce PD-L1-mediated immune evasion via integrin/BRAF/TAK1/ERK/ETV4 signaling [J]. Cancer Biology Med.

[59] HENRY K E, MACK K N NAGLEVL (2021). ERK Inhibition improves Anti-PD-L1 Immune Checkpoint Blockade in Preclinical Pancreatic Ductal Adenocarcinoma [J]. Mol Cancer Ther.

