# Supplementary material for: Clinical significance of low expression of CADM3 in breast cancer and preliminary exploration of related mechanisms
Source: BMC Cancer. 2024 Mar 21;24:367. doi: 10.1186/s12885-024-12114-y (PMC10958964; doi:10.1186/s12885-024-12114-y)
Supplement: Supplementary file 1 — Supplementary Material 1 [file 12885_2024_12114_MOESM1_ESM.docx]

Supplementary table1 The sequences of CADM3 and its control for RNA transfection.

| ID | seq |
| --- | --- |
| CADM3(80262-11)-p1 | CCAACTTTGTGCCAACCGGTCGCCACCATGGGGGCCCCAGCCGCCTC |
| CADM3(80262-11)-p2 | AATGCCAACTCTGAGCTTGATGAAATATTCCTTCTTGTCGTC |

Supplementary figure1 Protein expression of CADM3 in 8 cases of human BC and its adjacent pairs of normal tissues. (Full length gels and blots of figure 1E.)

a. CADM3 expression in the 1st pair of tissues.


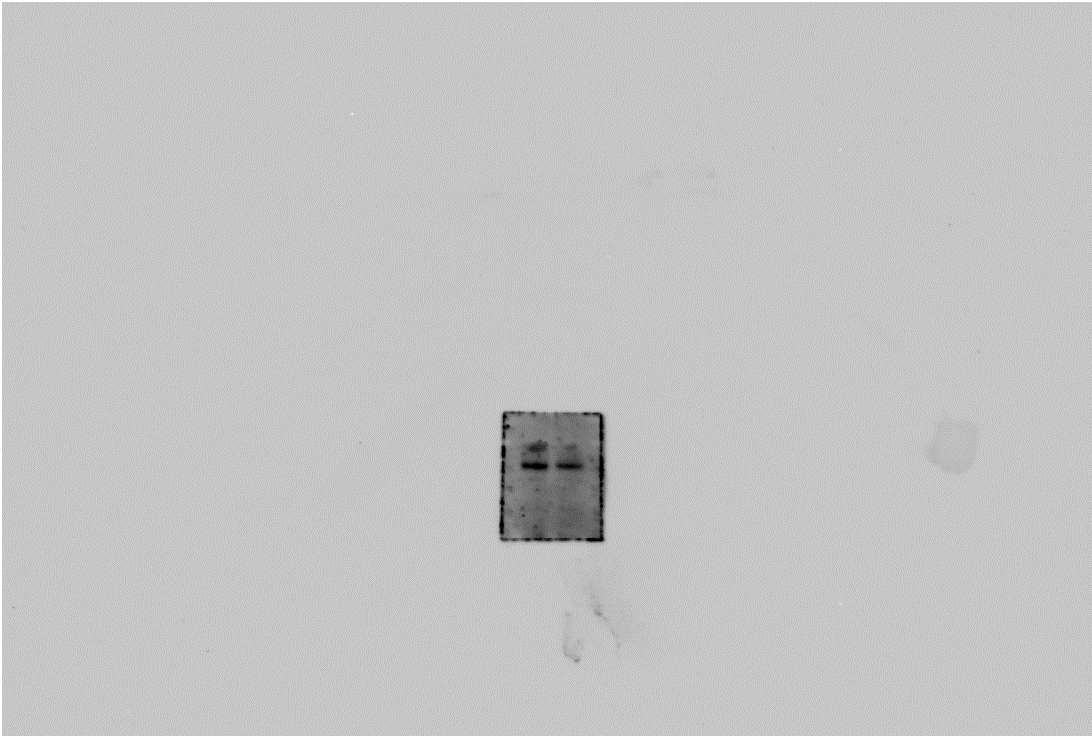


b. GAPDH expression in the 1st pair of tissues.


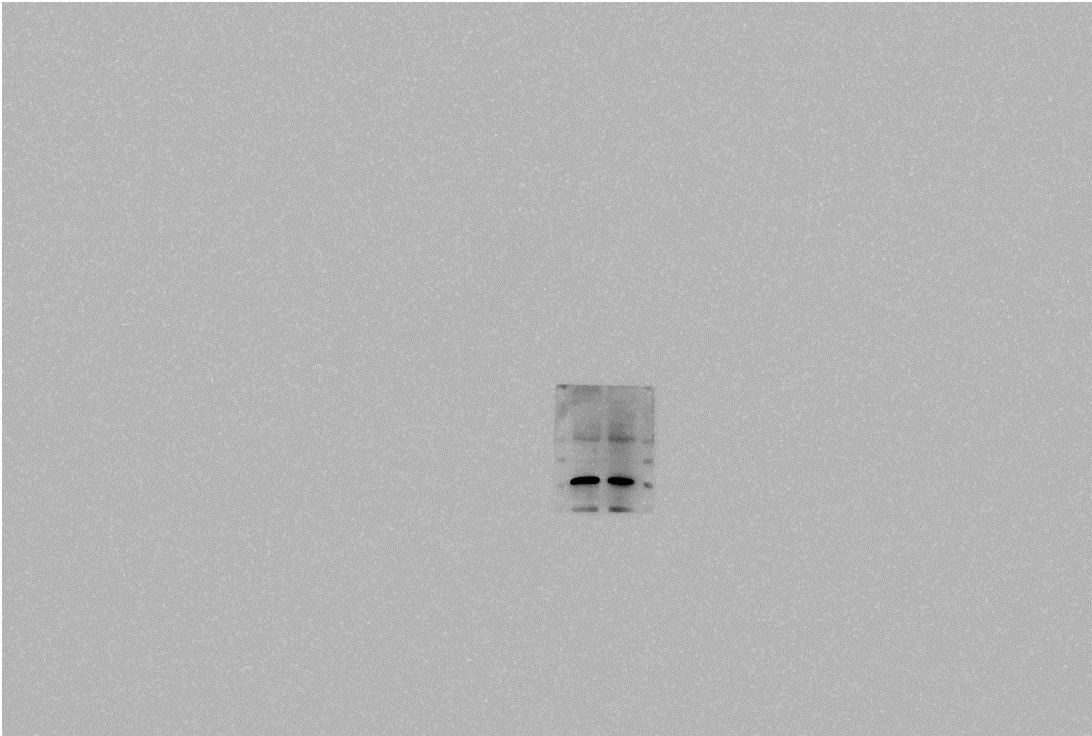


c. CADM3 expression in the 2nd pair of tissues.


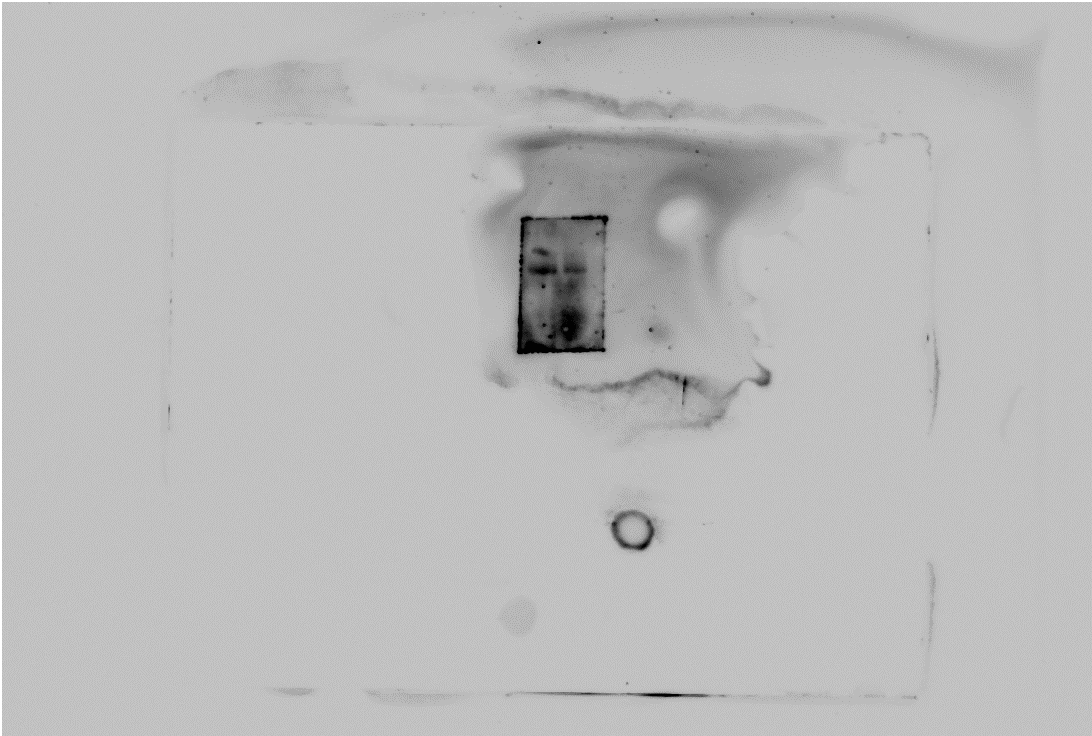


d. GAPDH expression in the 2st pair of tissues.


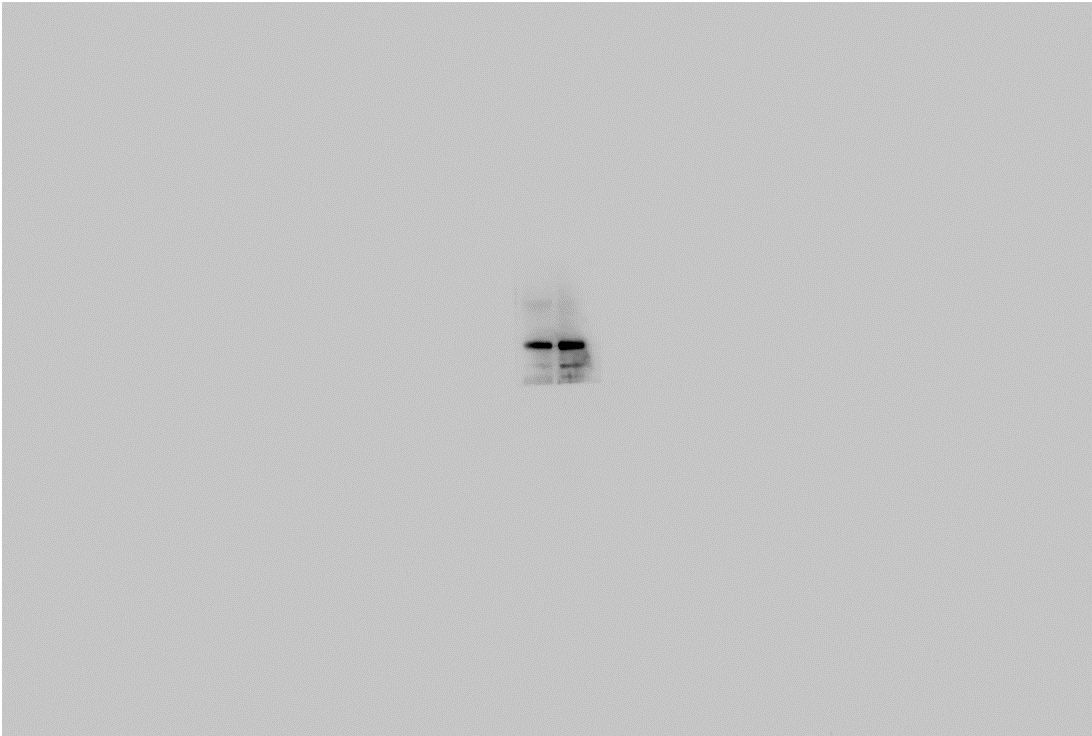


e. CADM3 expression in the 3rd pair of tissues.


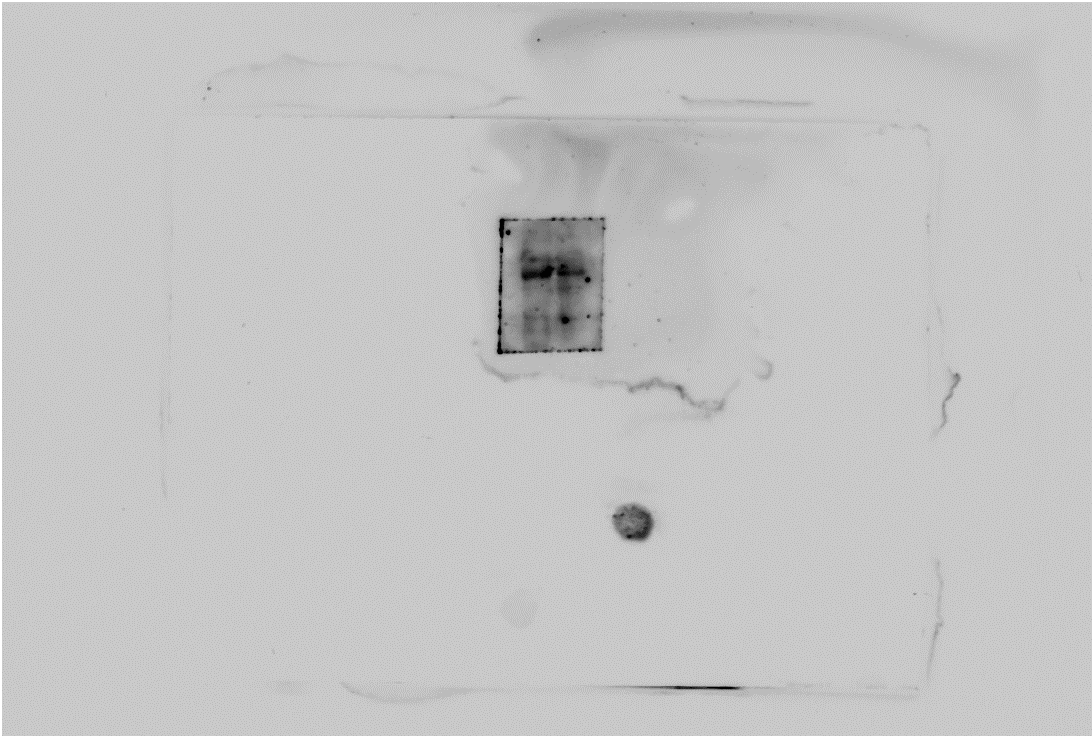


f. GAPDH expression in the 3rd pair of tissues.


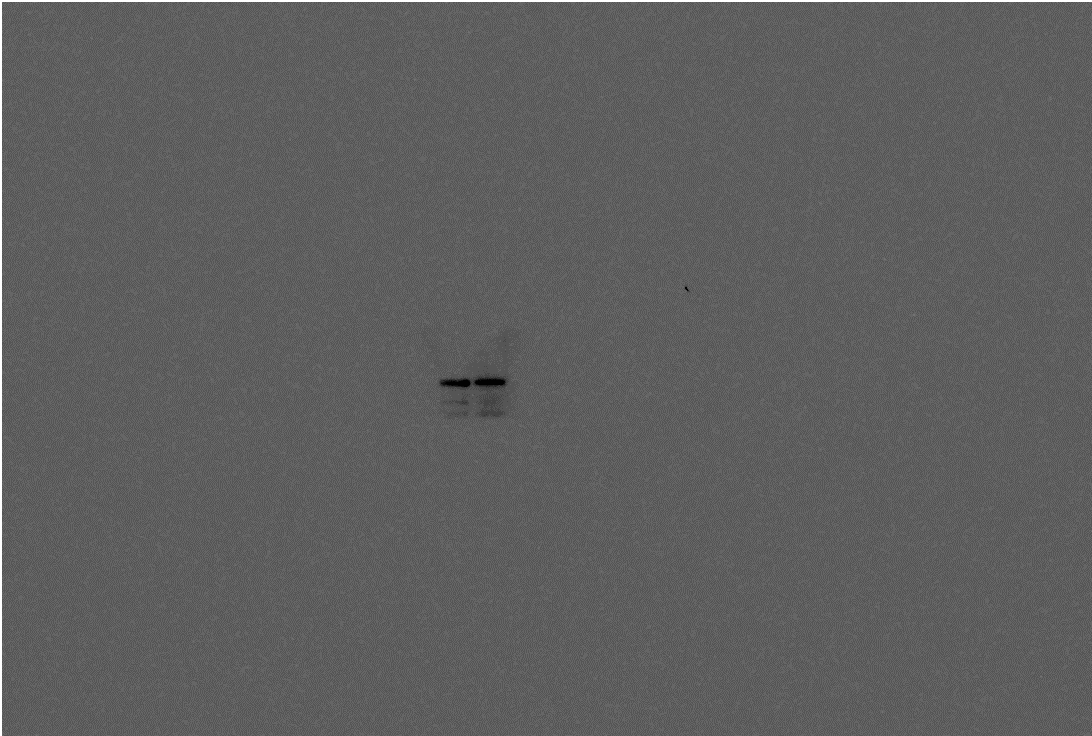


g. CADM3 expression in the 4th pair of tissues.


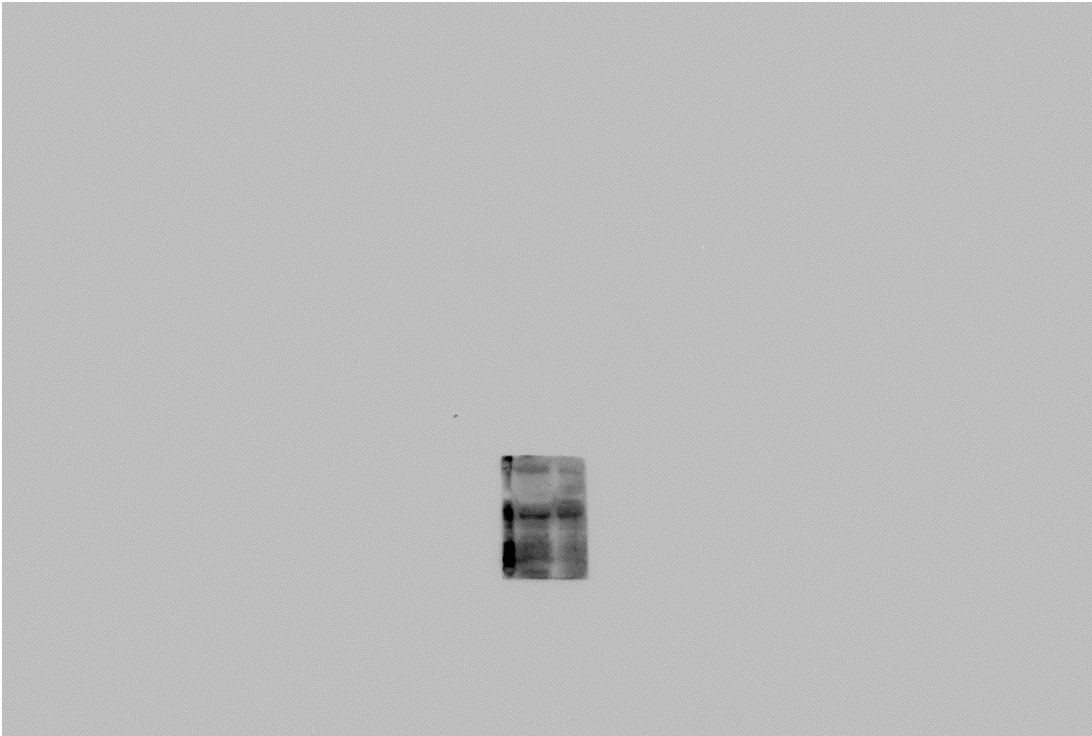


h. GAPDH expression in the 4th pair of tissues.


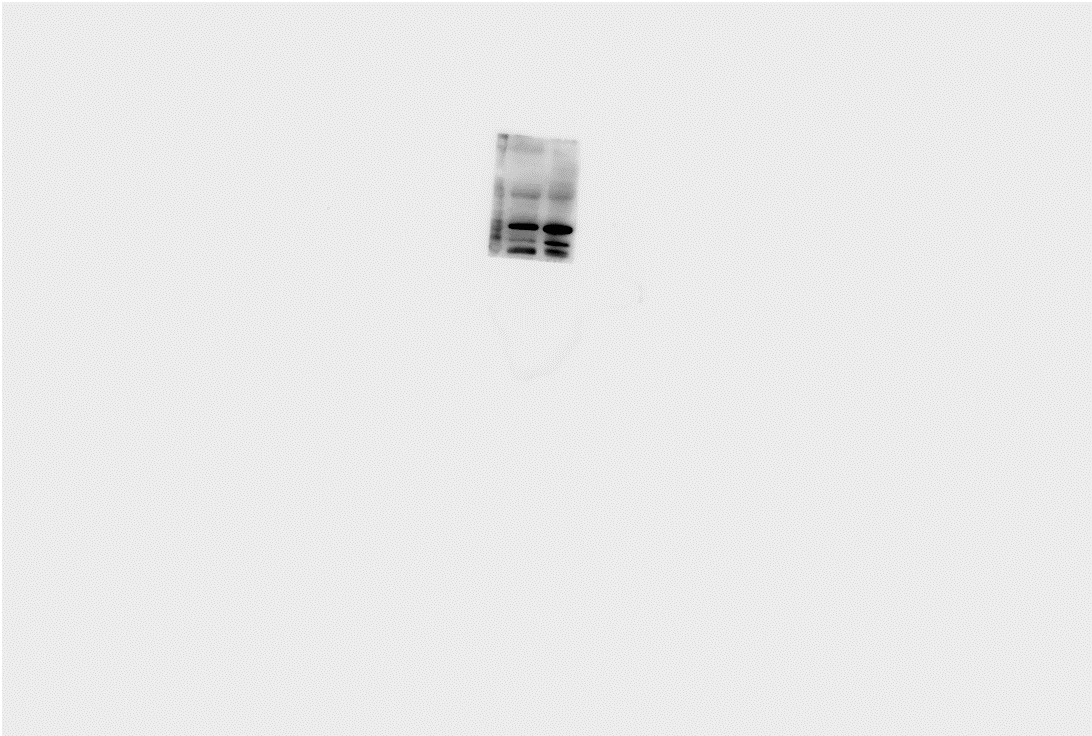


i. CADM3 expression in the 5th pair of tissues.


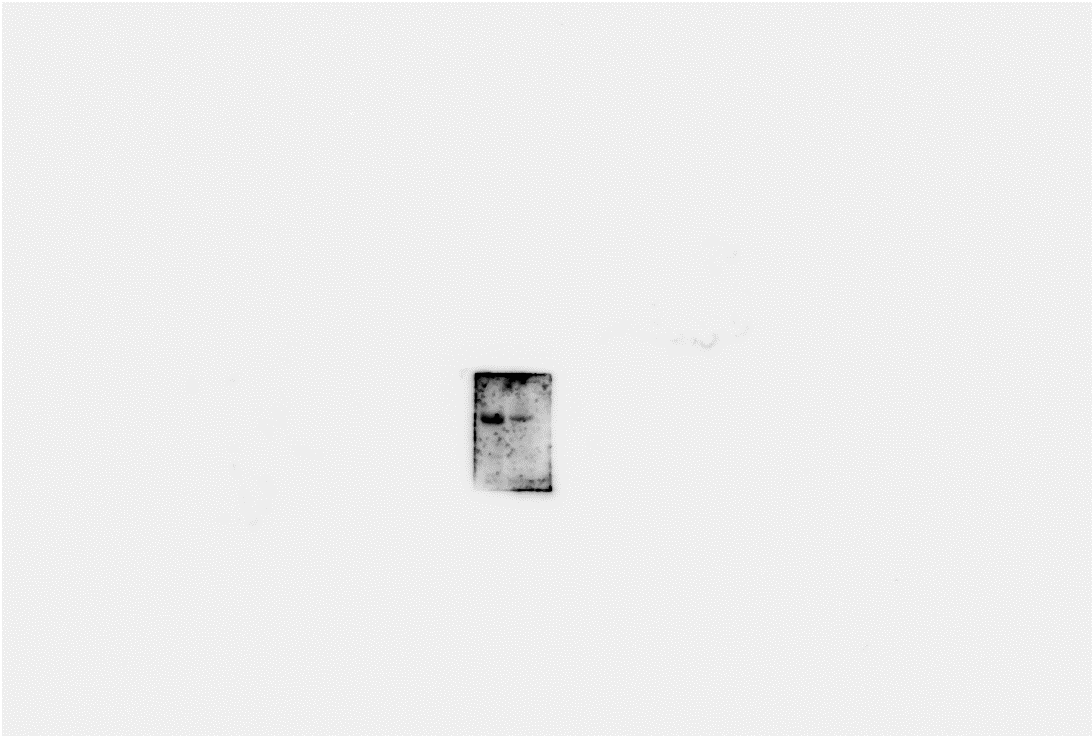


j. GAPDH expression in the 5th pair of tissues.


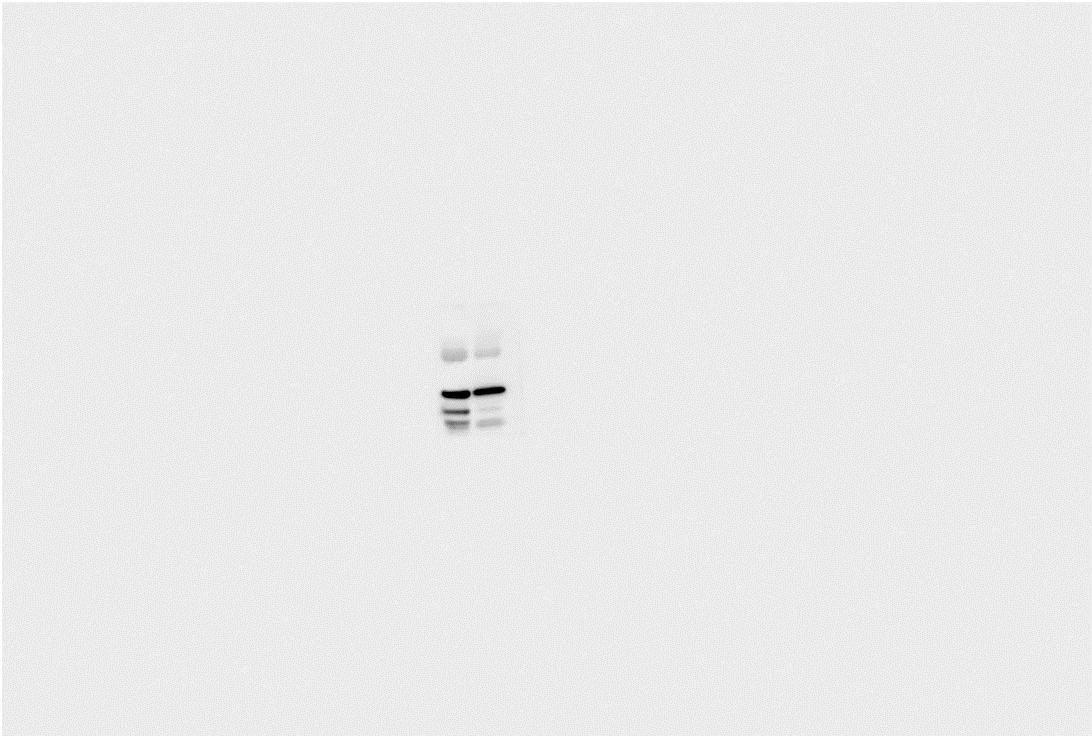


k. CADM3 expression in the 6th pair of tissues.


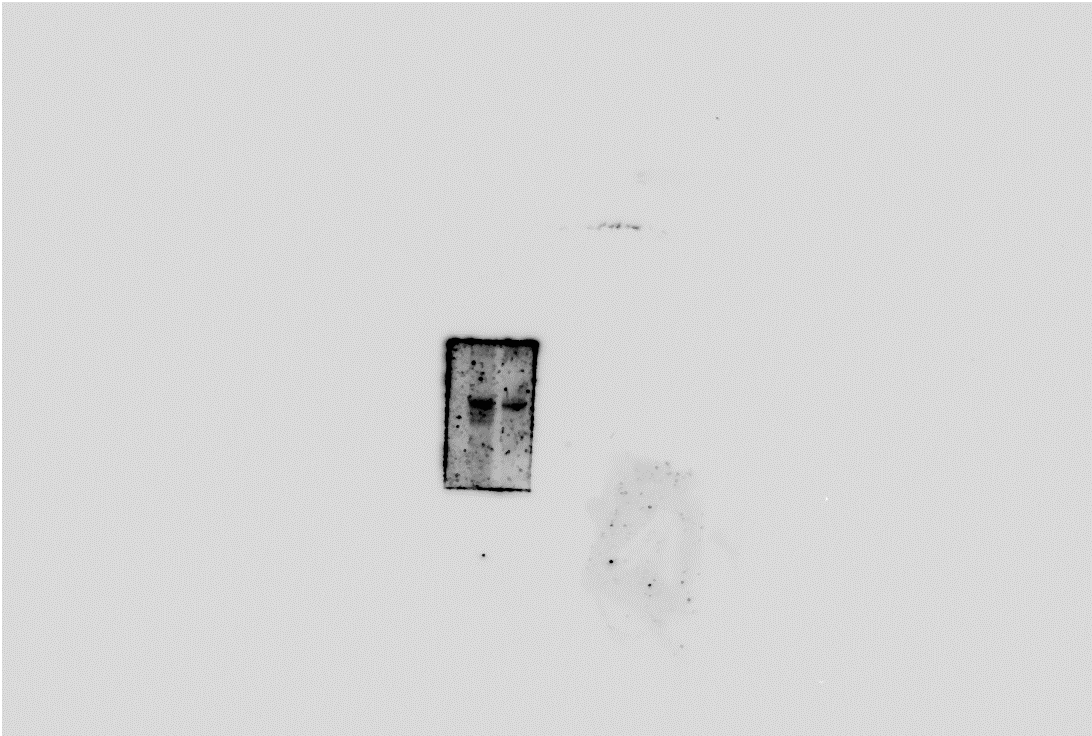


l. GAPDH expression in the 6th pair of tissues.


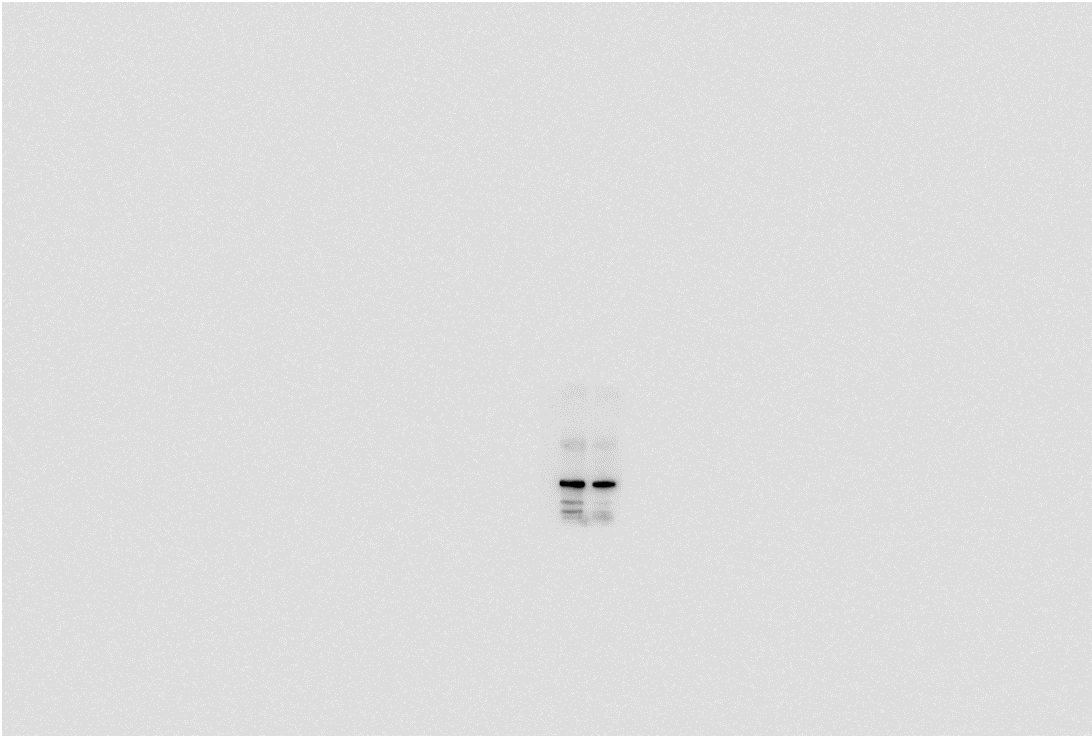


m. CADM3 expression in the 7th pair of tissues.


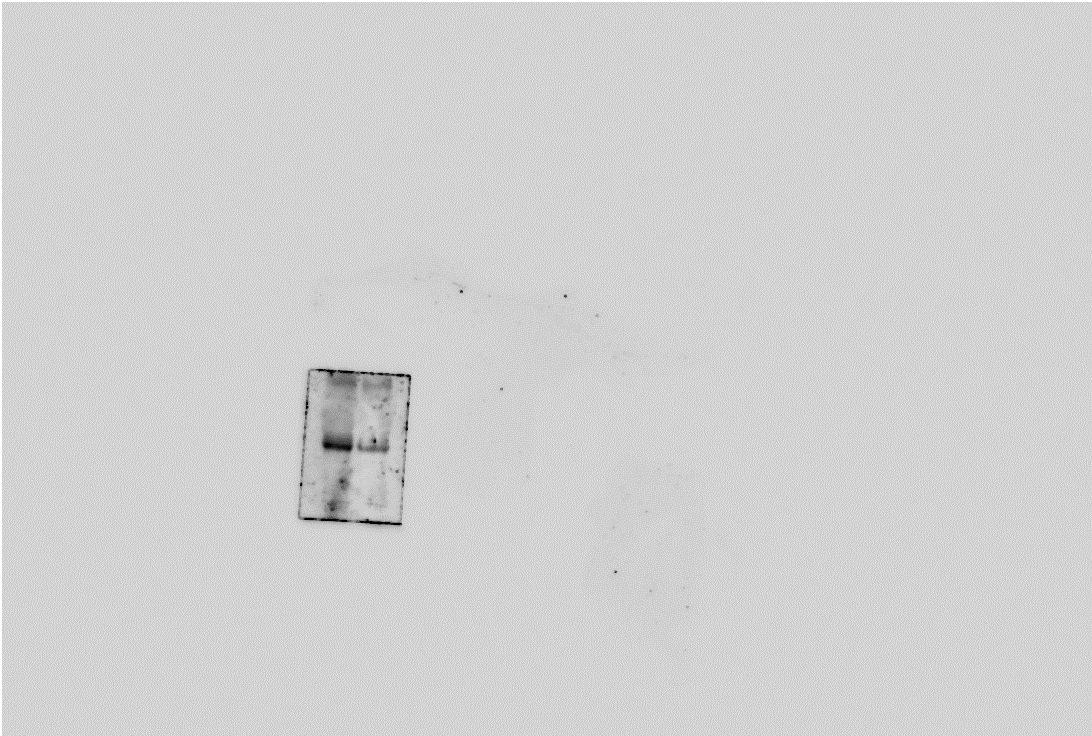


n, GAPDH expression in the 7th pair of tissues.


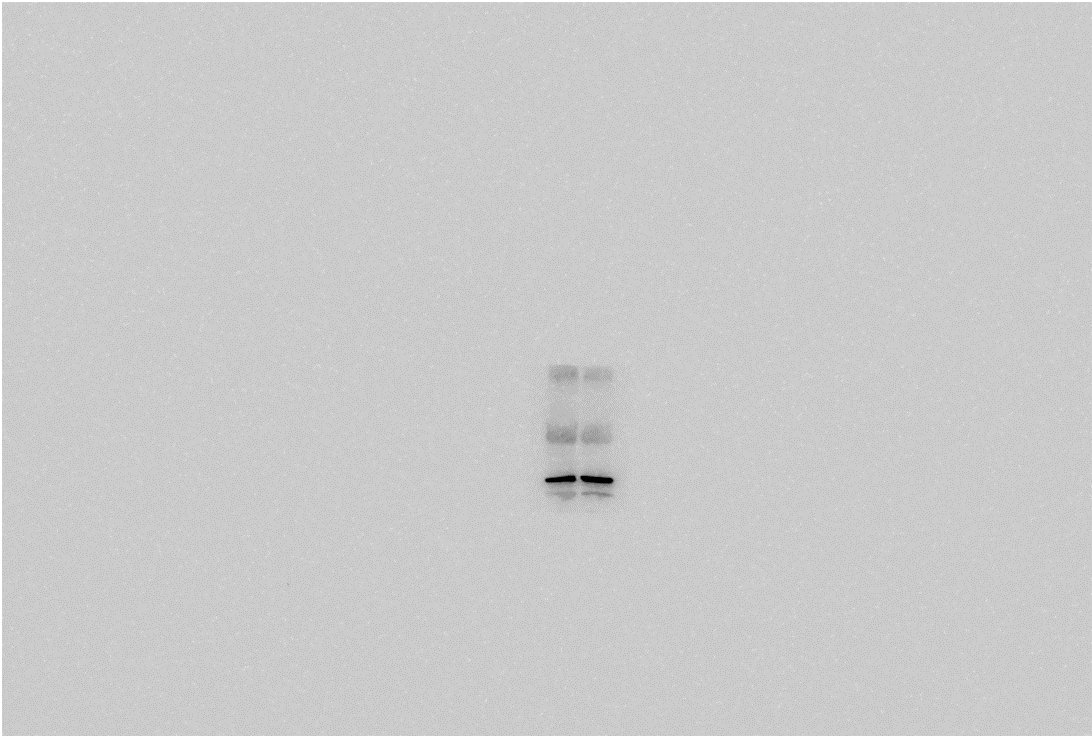


o. CADM3 expression in the 8th pair of tissues.


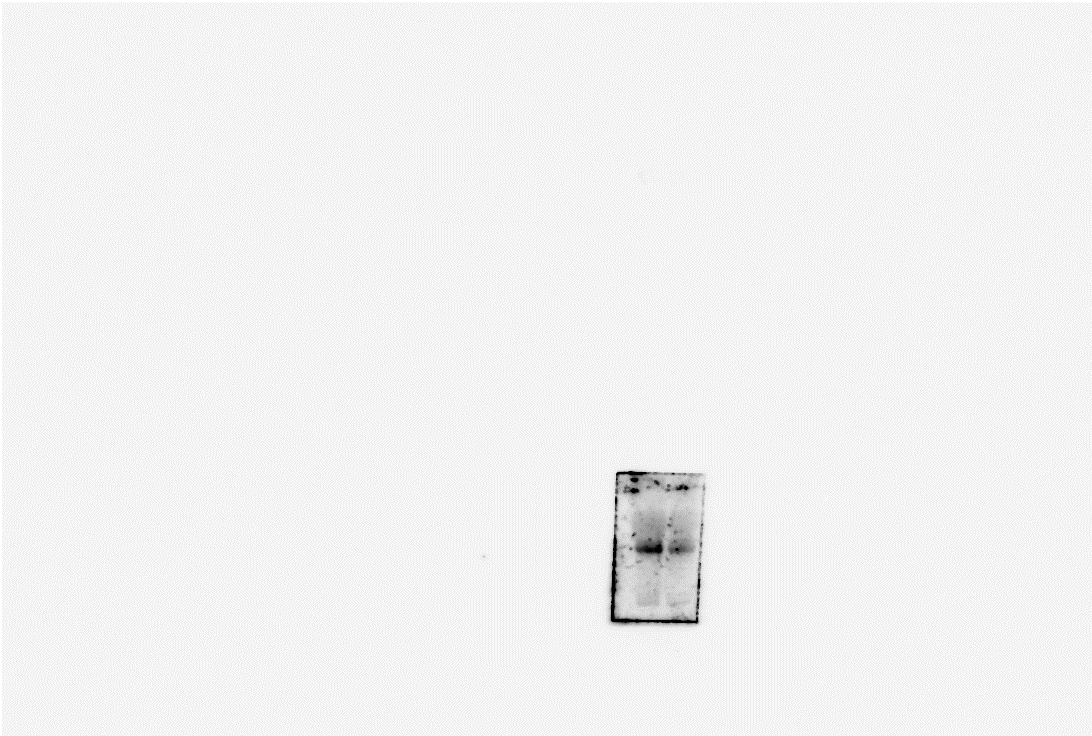


p. GAPDH expression in the 8th pair of tissues.


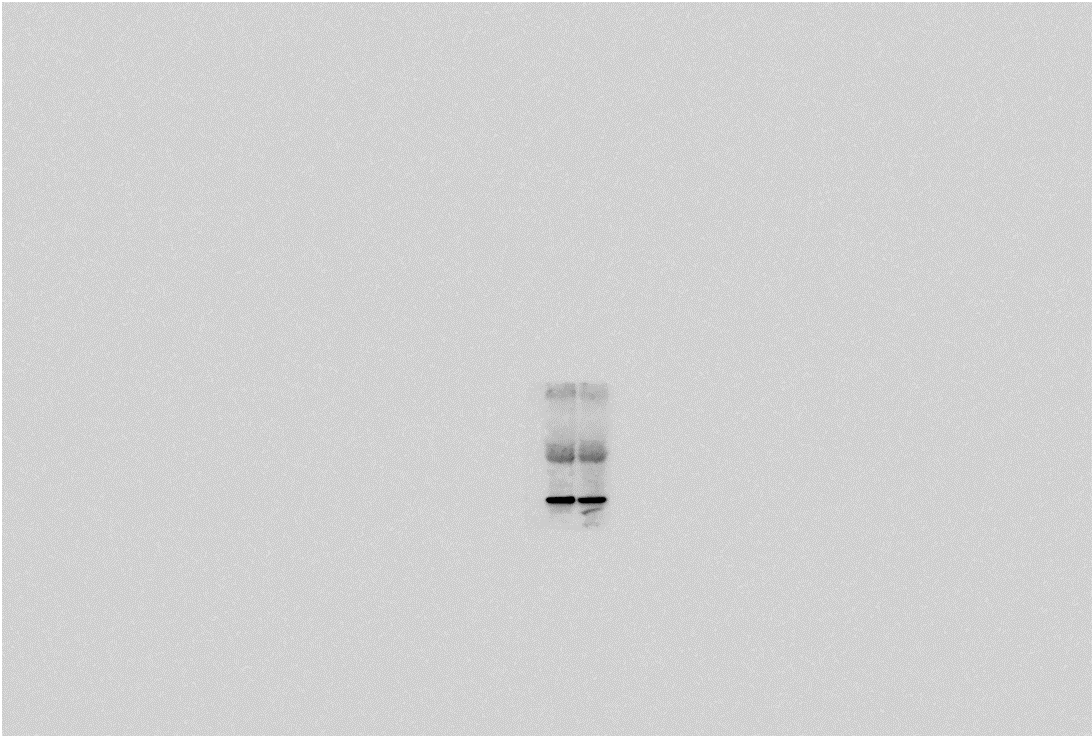


Supplementary figure2 The overexpression efficiency of CADM3 in MCF-7 and MDA-MB-231 cell lines verified by WB. (Full length gels and blots of figure 5A.)

a. CADM3


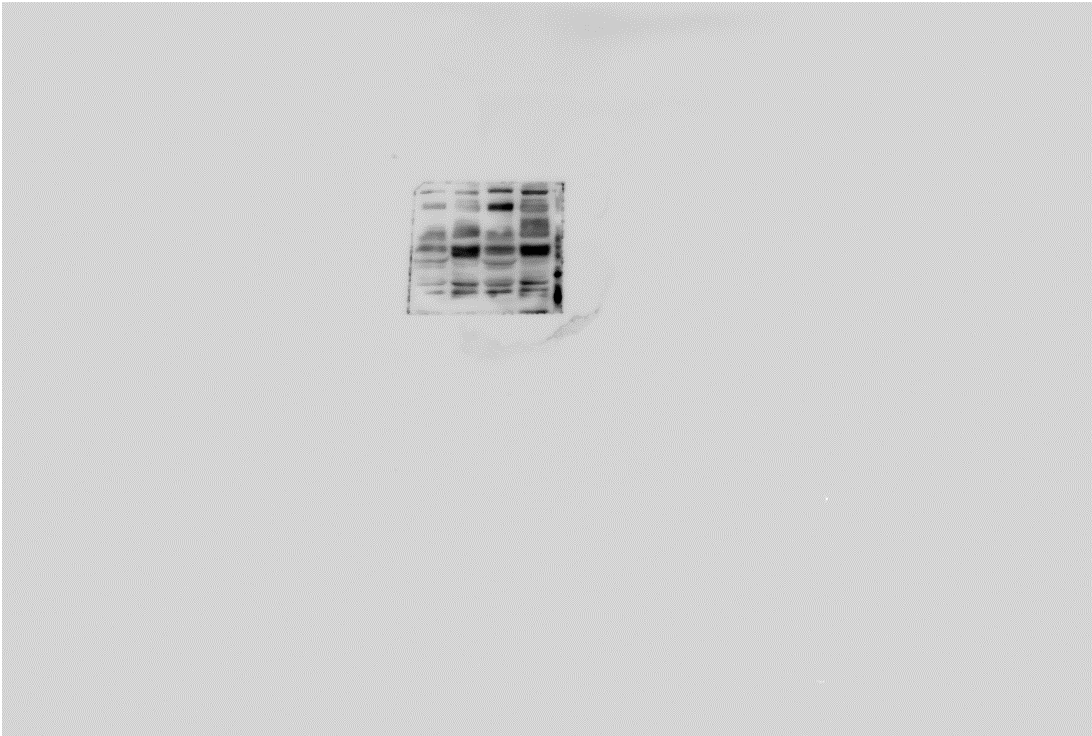


b.GAPDH


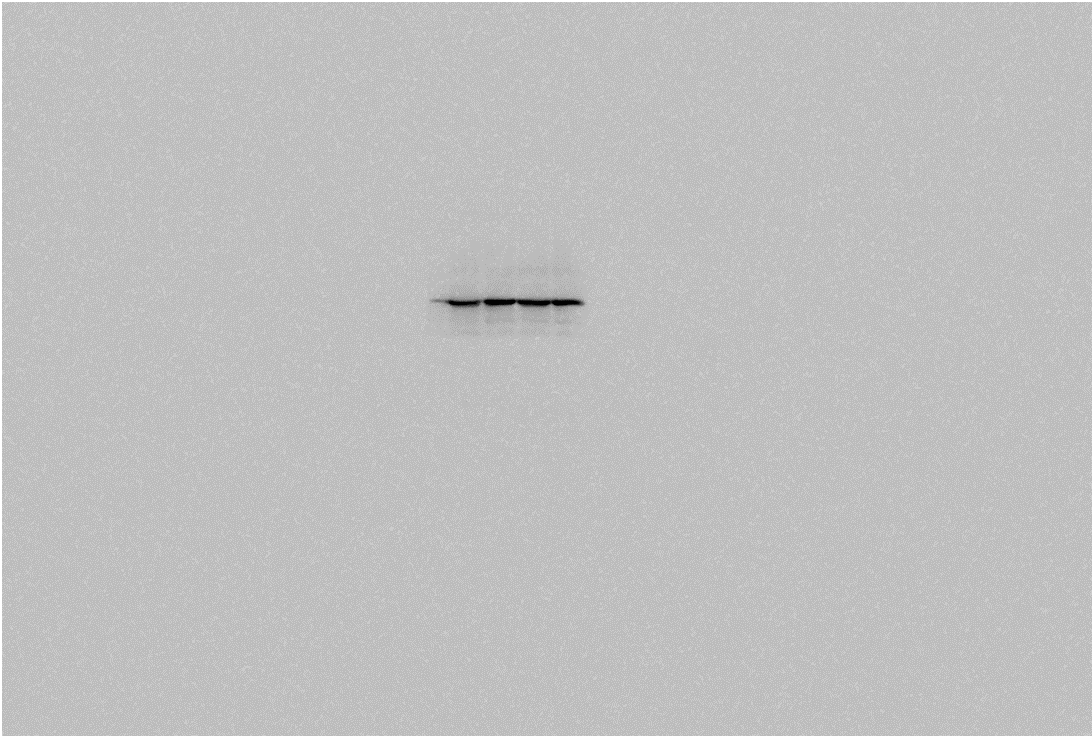


Supplementary figure3 The expressions of key proteins P38, ERK, JNK and their phosphorylated proteins in MAPK pathway were verified by WB assay. (Full length gels and blots of figure 8.)

a.MCF-7 P38（CADM3）


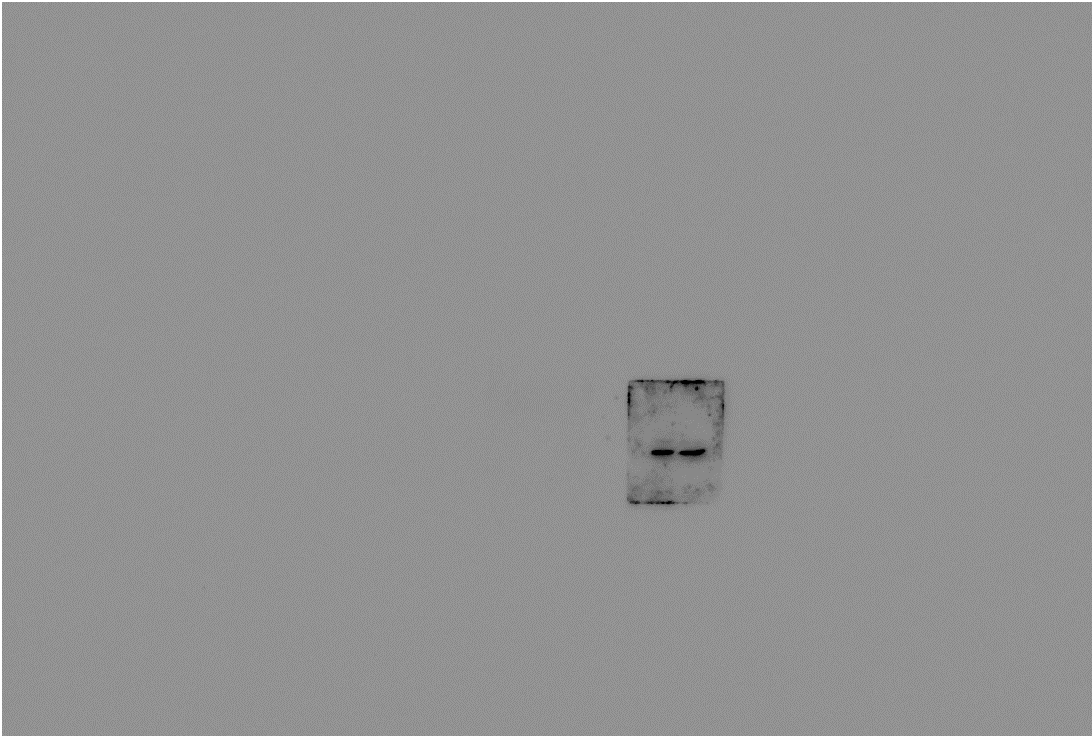


b.MCF-7 P38（GAPDH）


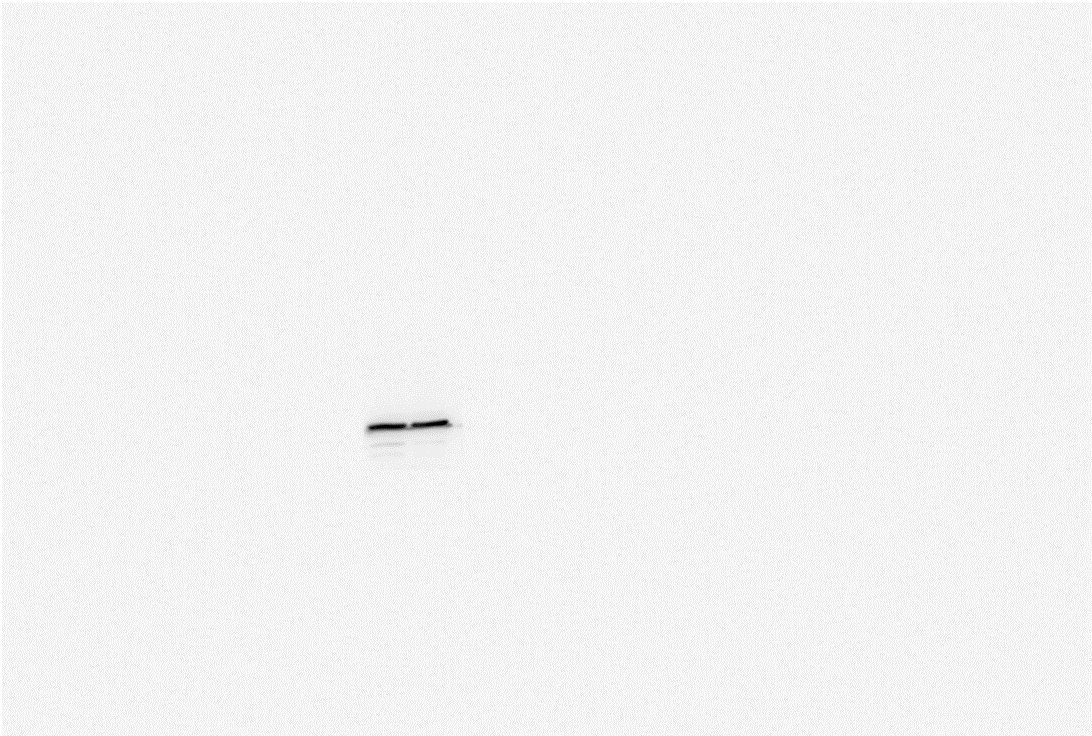


c.MCF7 P-P38（CADM3）


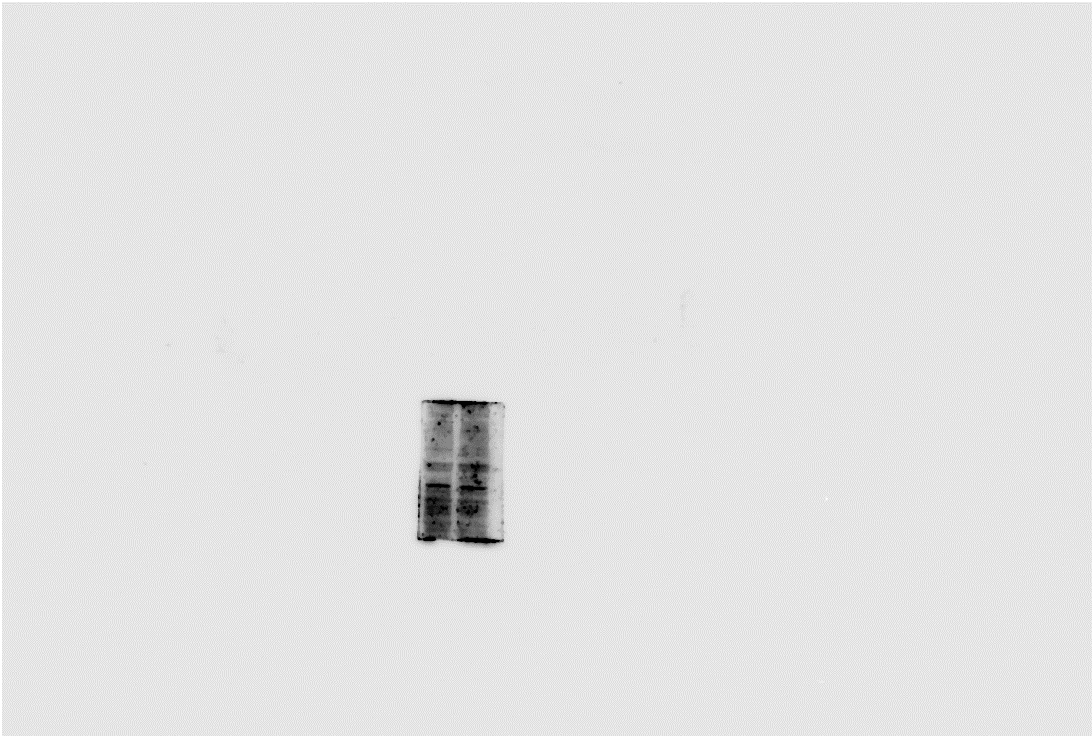


d.MCF7 P-P38（GAPDH）


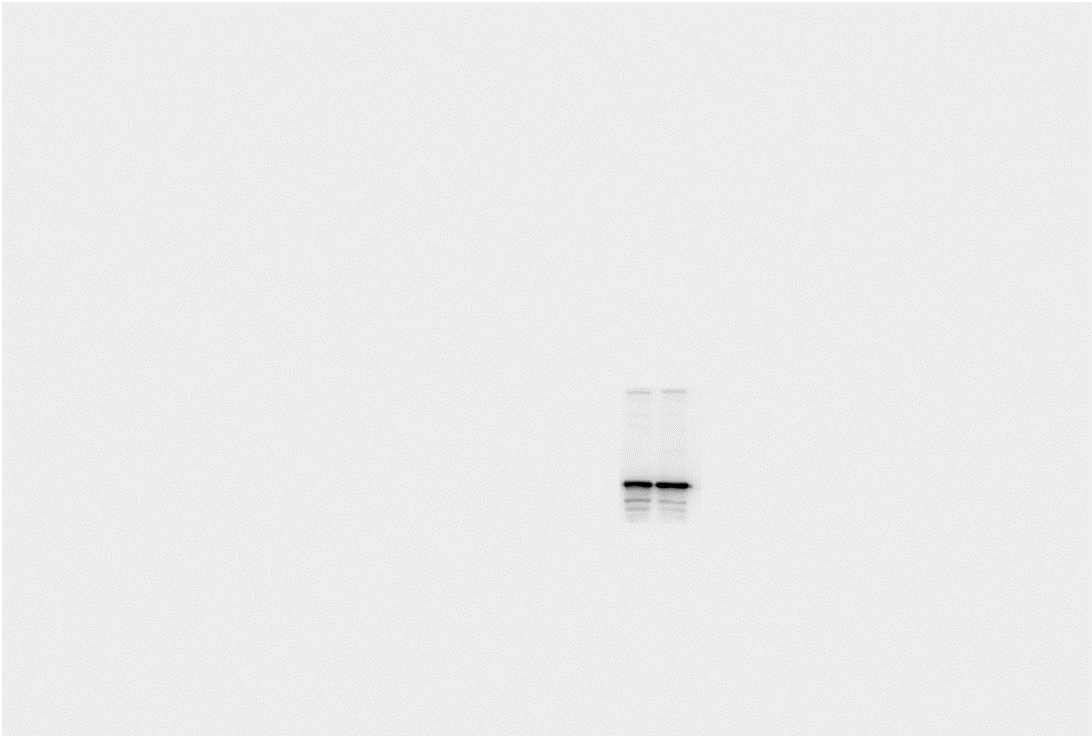


e.MCF-7 ERK (CADM3)


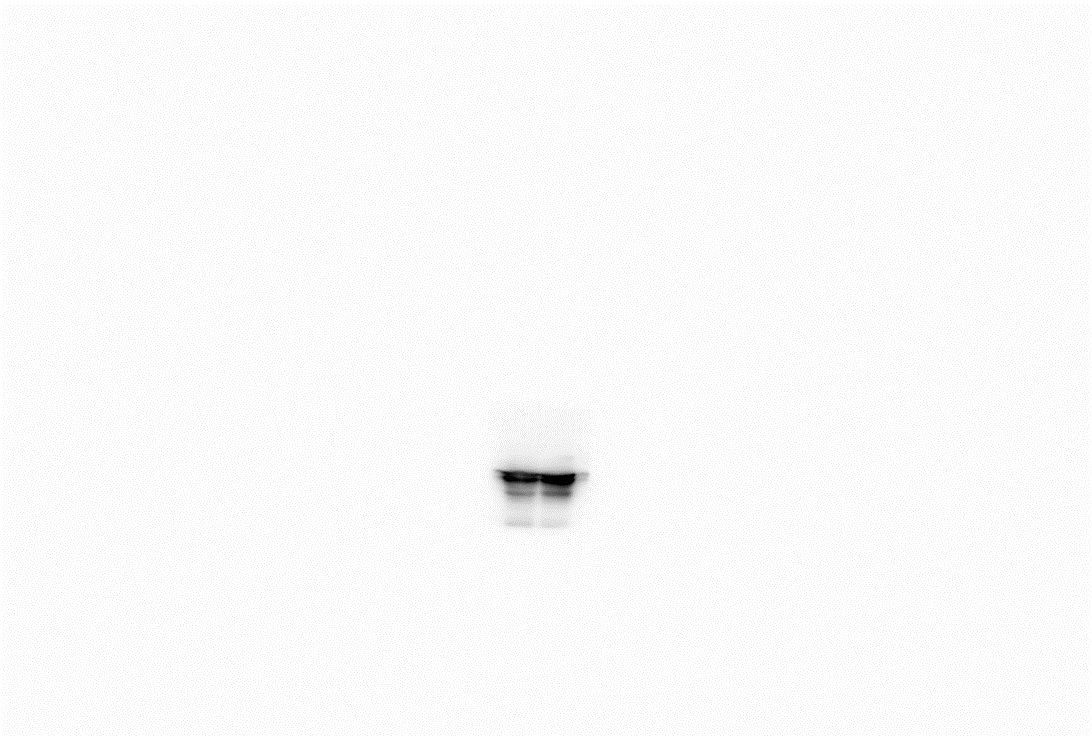


f.MCF-7 ERK (GAPDH)


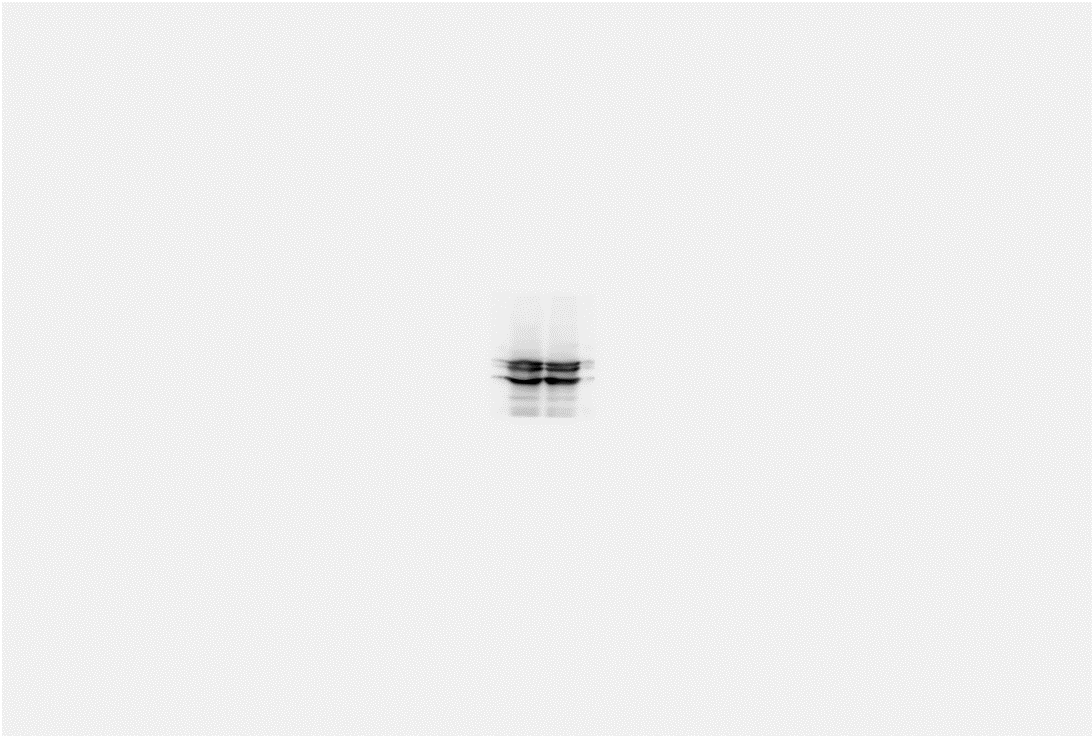


g.MCF-7 P-ERK (CADM3)


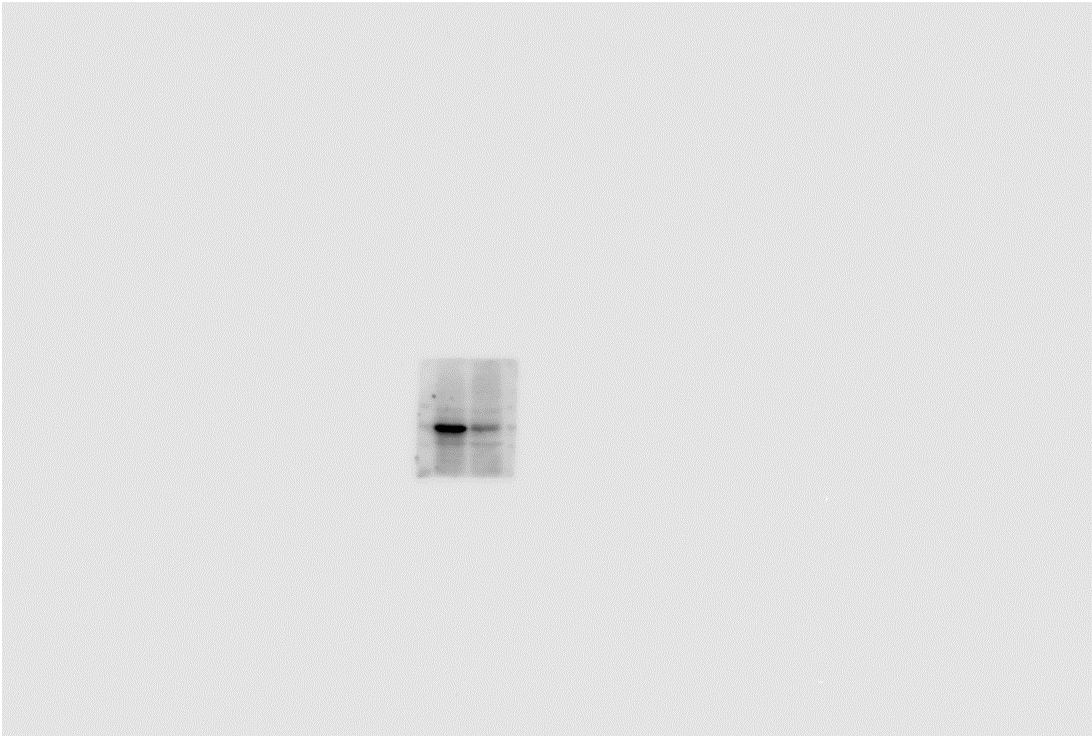


h. MCF-7 P-ERK (GAPDH)


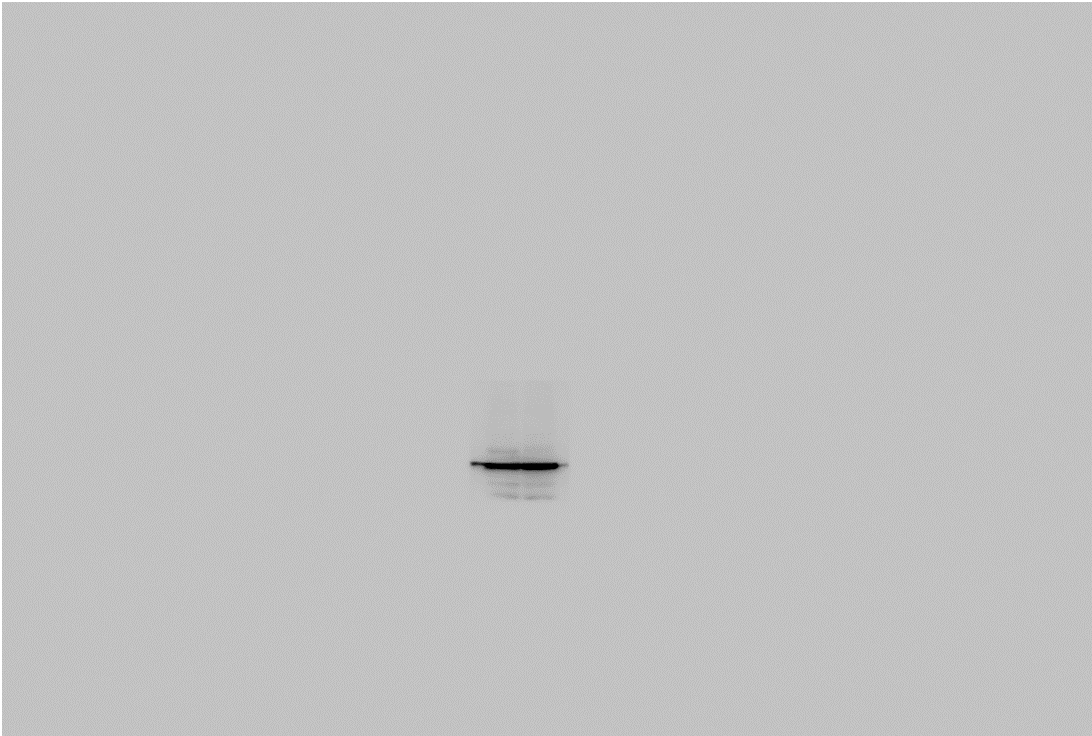


i.MCF-7 JNK (CADM3)


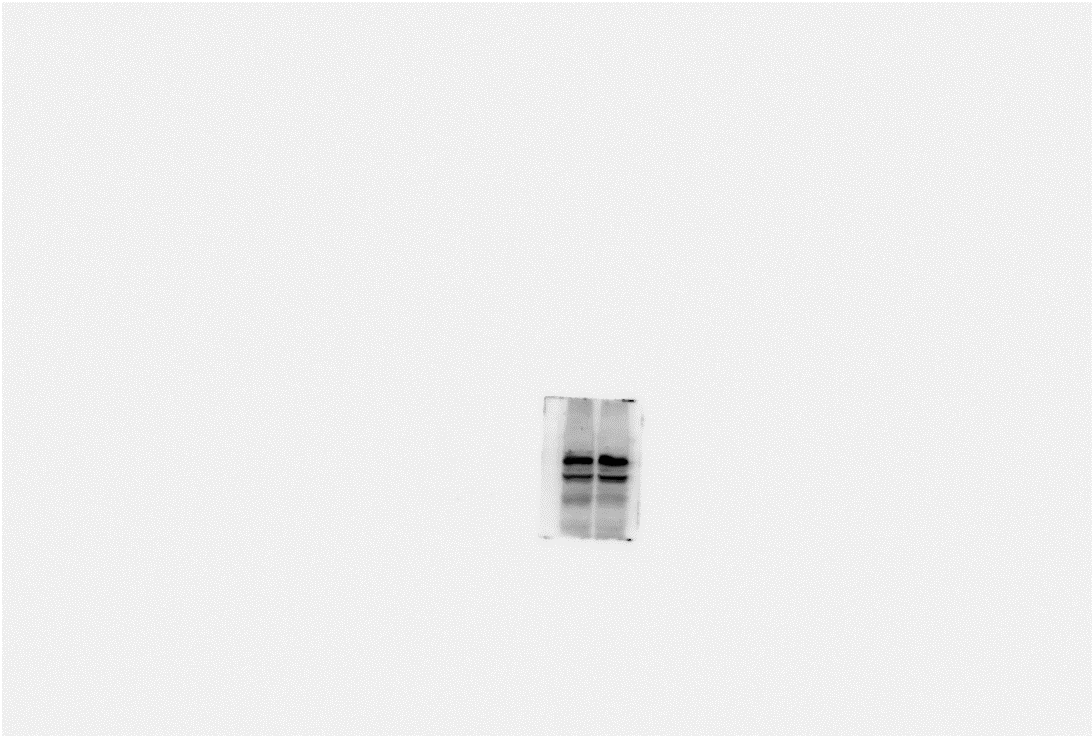


j. MCF-7 JNK (GAPDH)


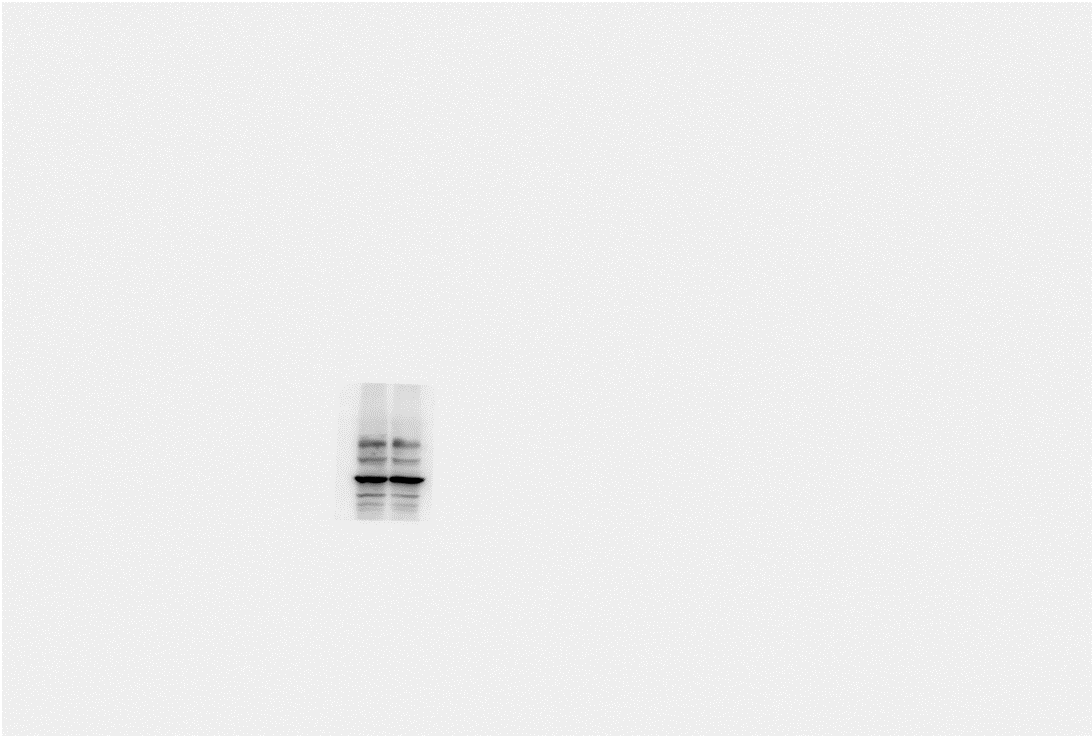


k.MCF-7 P-JNK (CADM3)


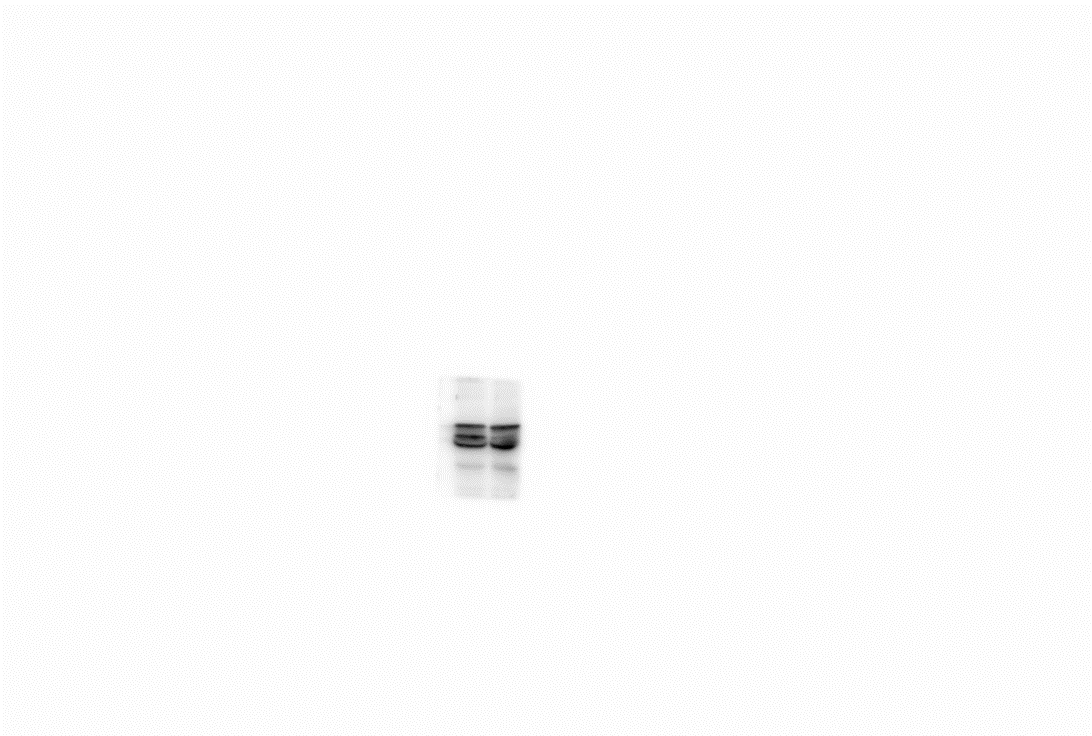


l. MCF-7 P-JNK (GAPDH)


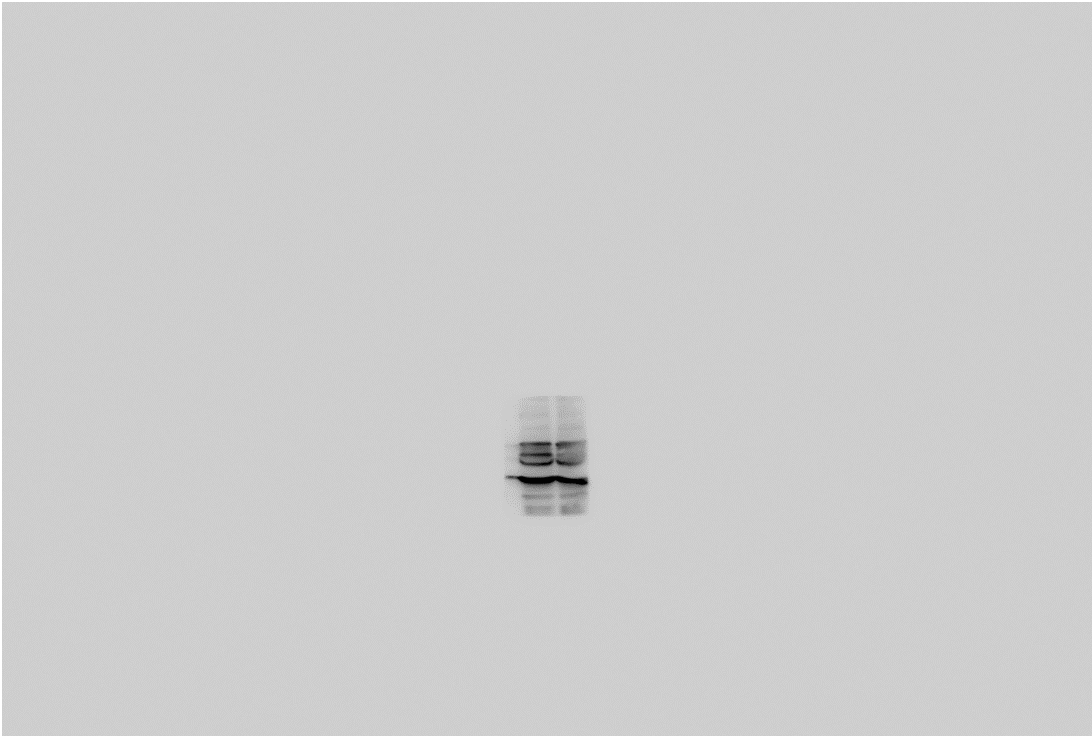


m.MDA-MB-231 P38 (CADM3)


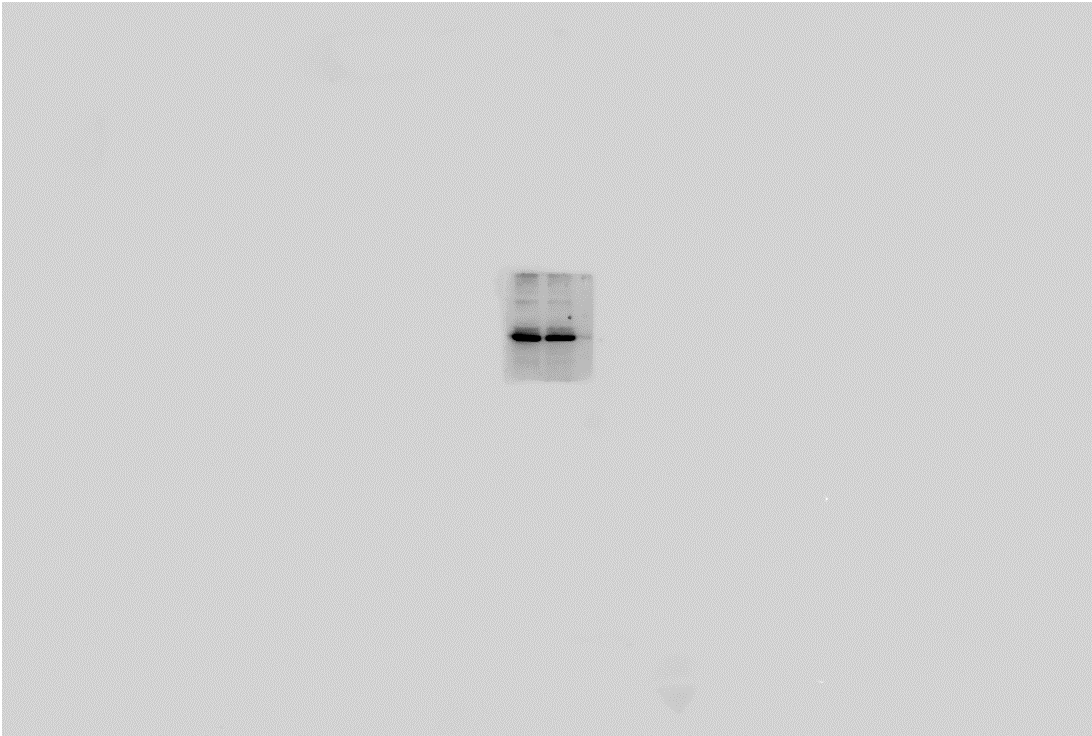


n. MDA-MB-231 P38 (GAPDH)


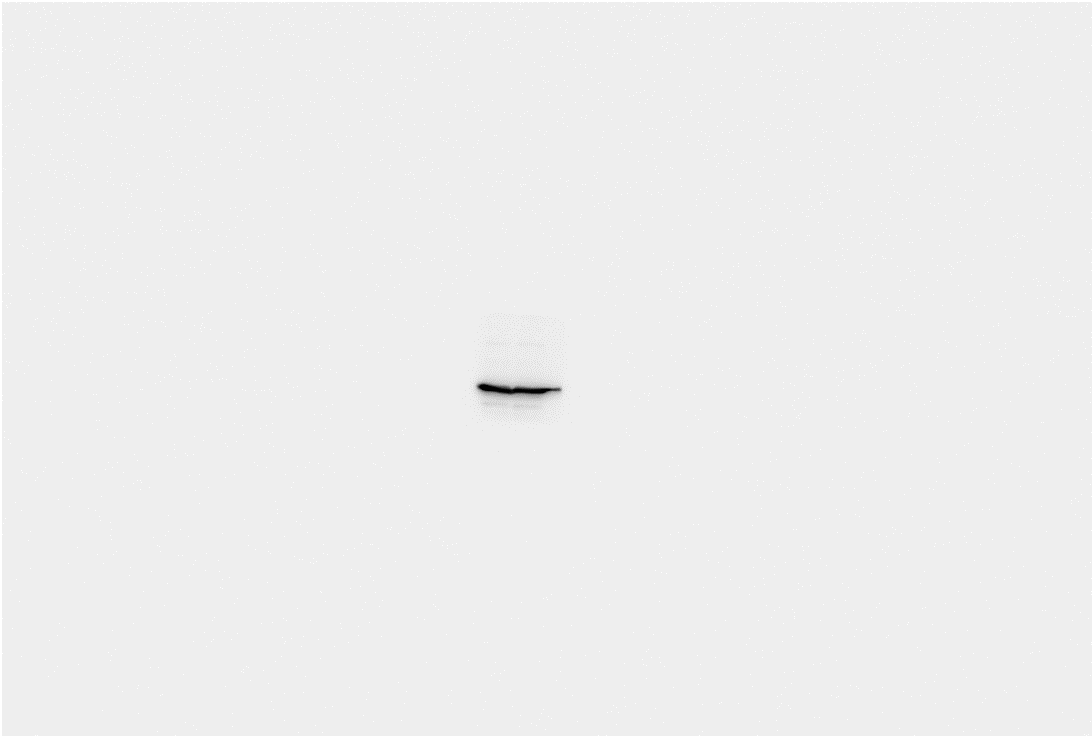


o.MDA-MB-231 P-P38 (CADM3)


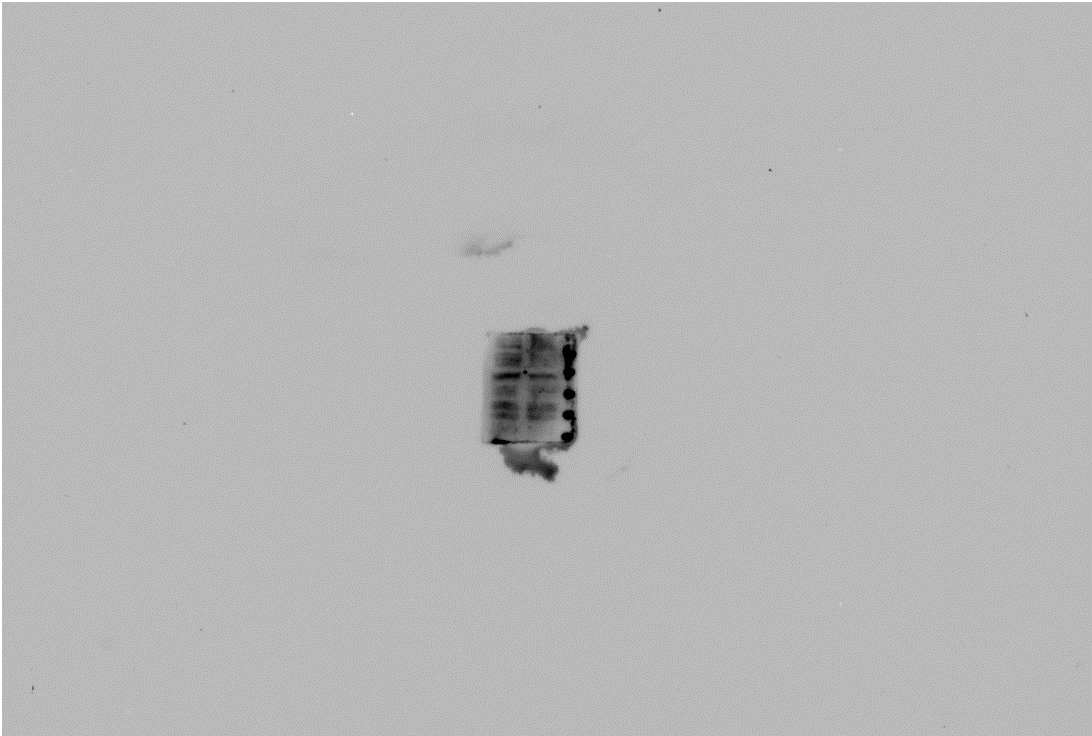


p. MDA-MB-231 P-P38 (GAPDH)


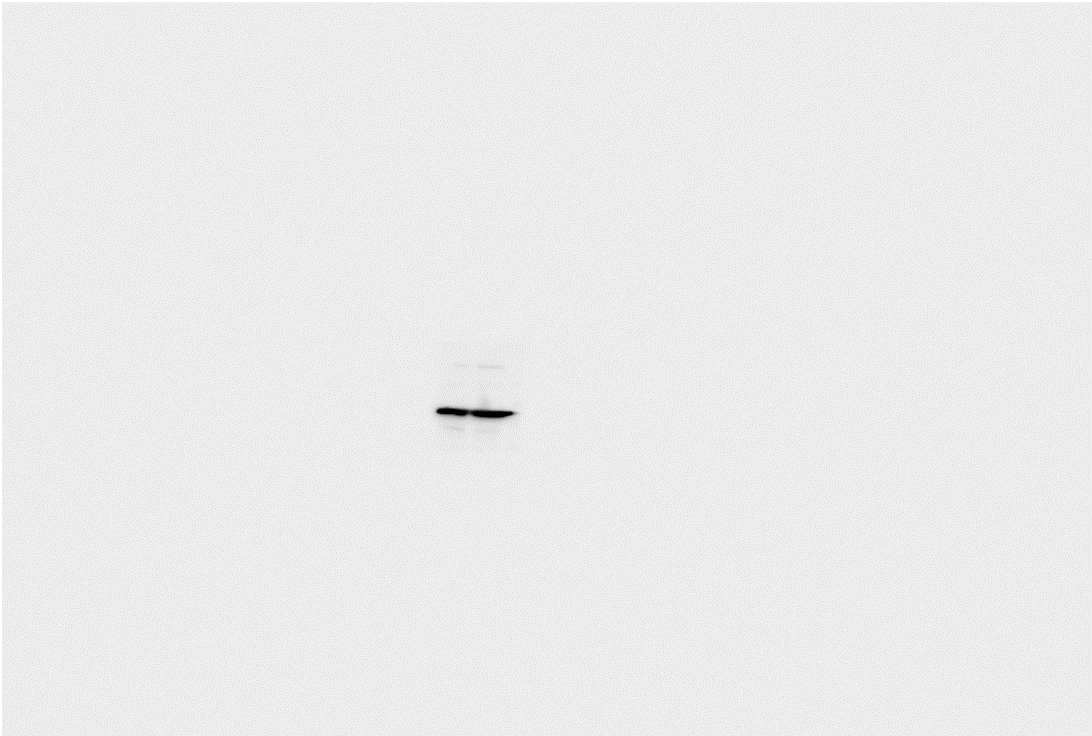


q.MDA-MB-231ERK (CADM3)


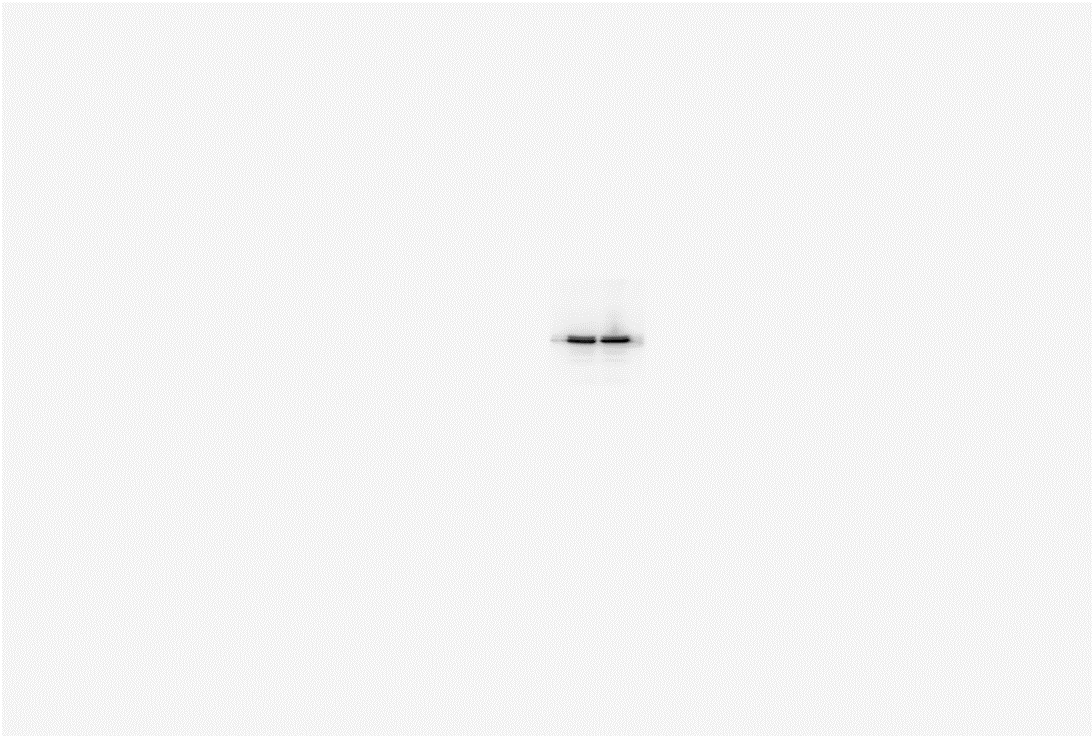


r. MDA-MB-231ERK (GAPDH)


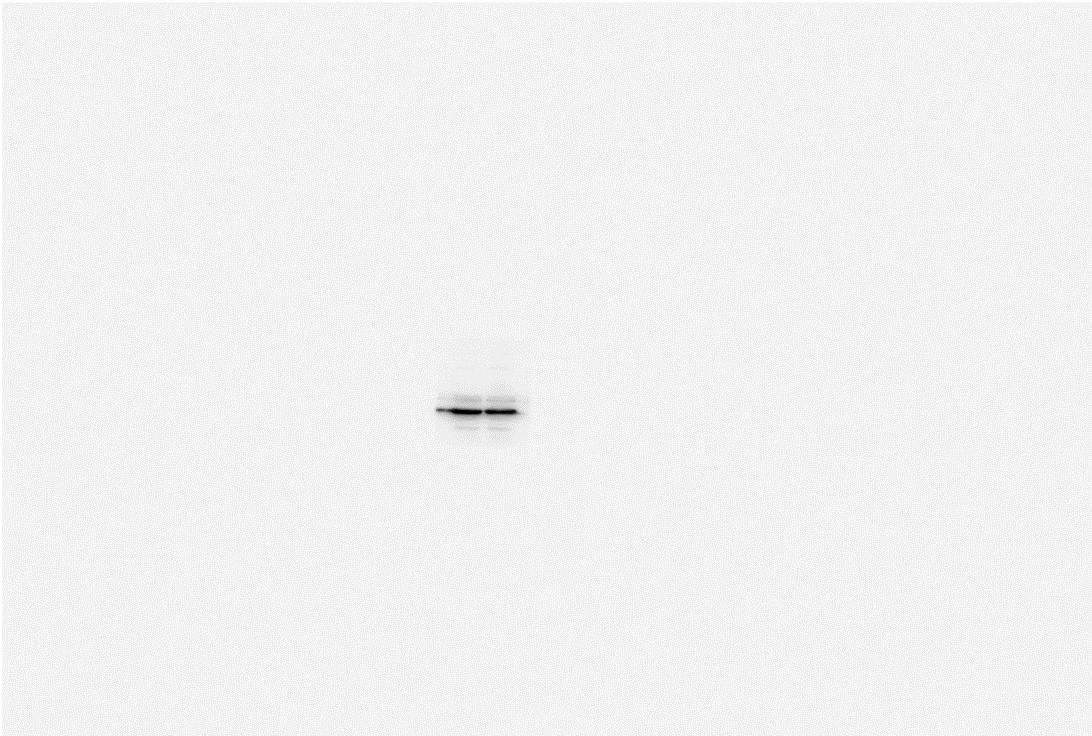


s.MDA-MB-231 P-ERK (CADM3)


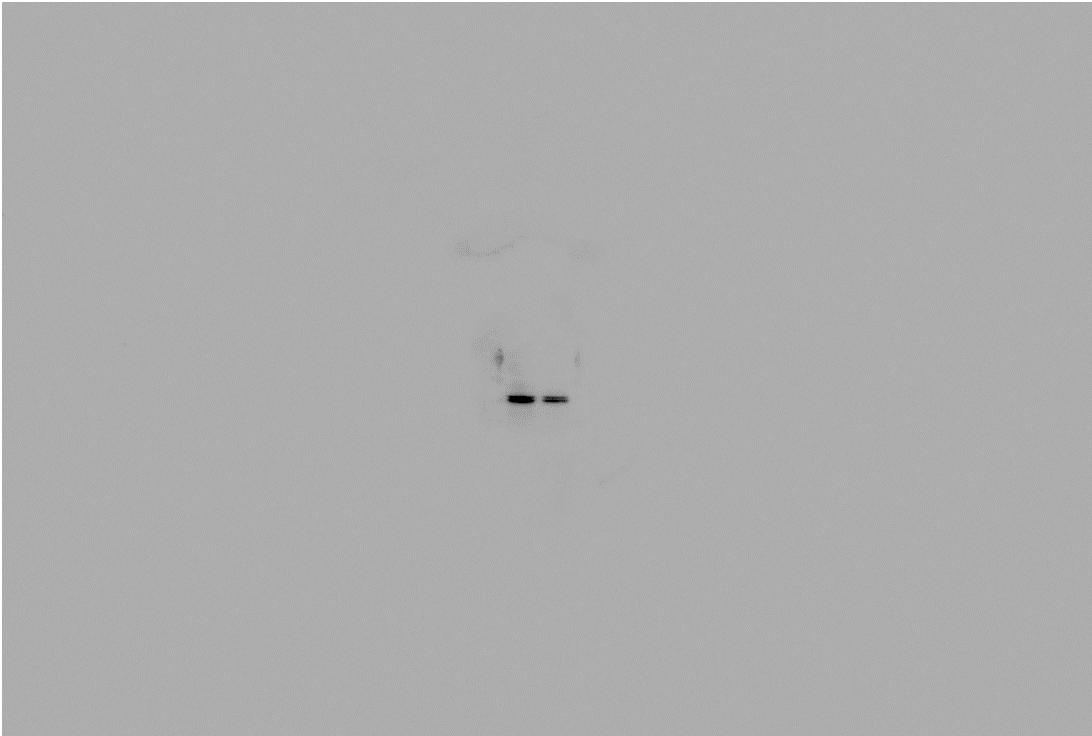


t. MDA-MB-231 P-ERK (GAPDN)


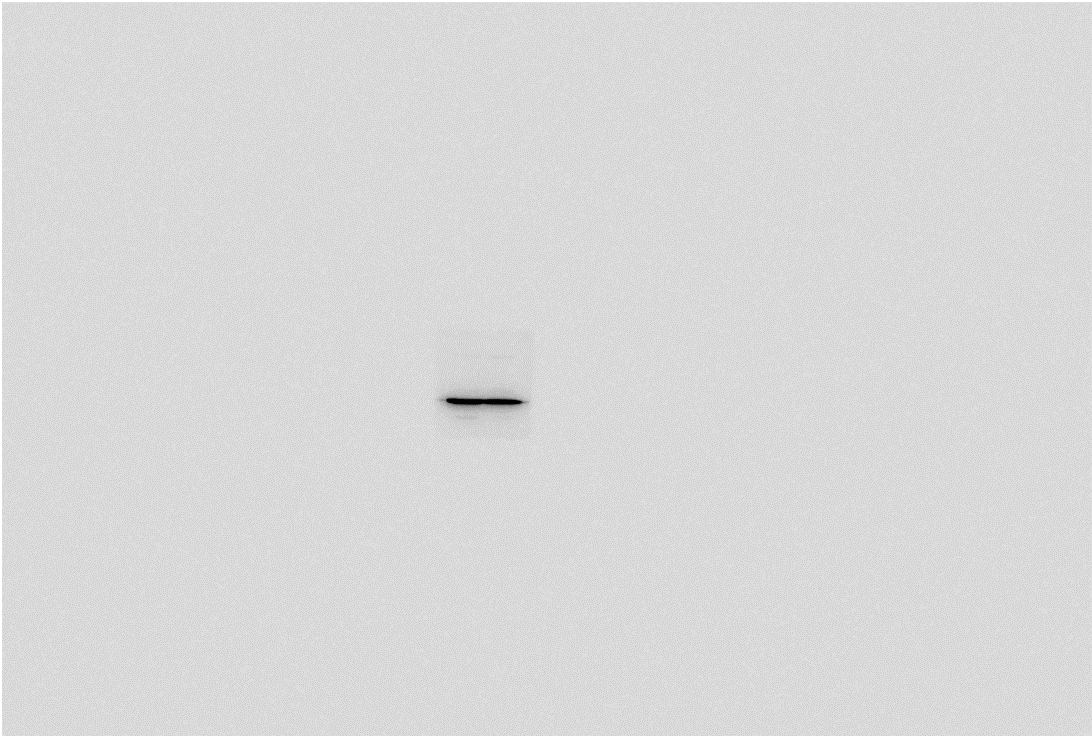


u.MDA-MB-231 JNK (CADM3)


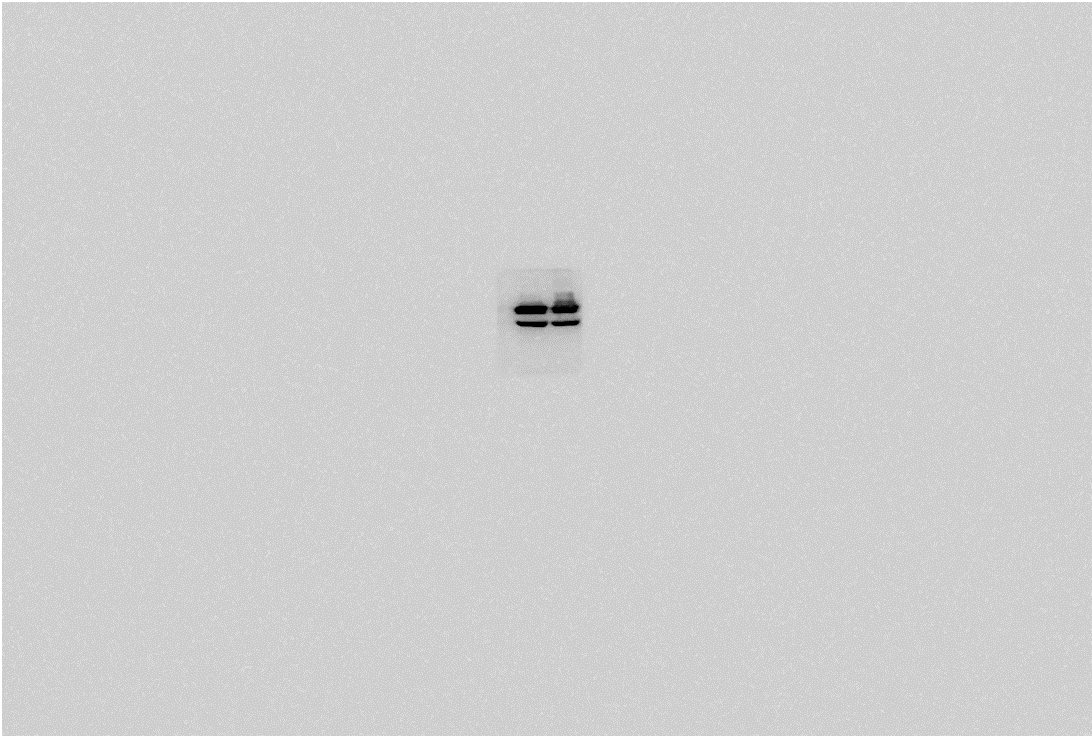


v. MDA-MB-231 JNK (GAPDH)


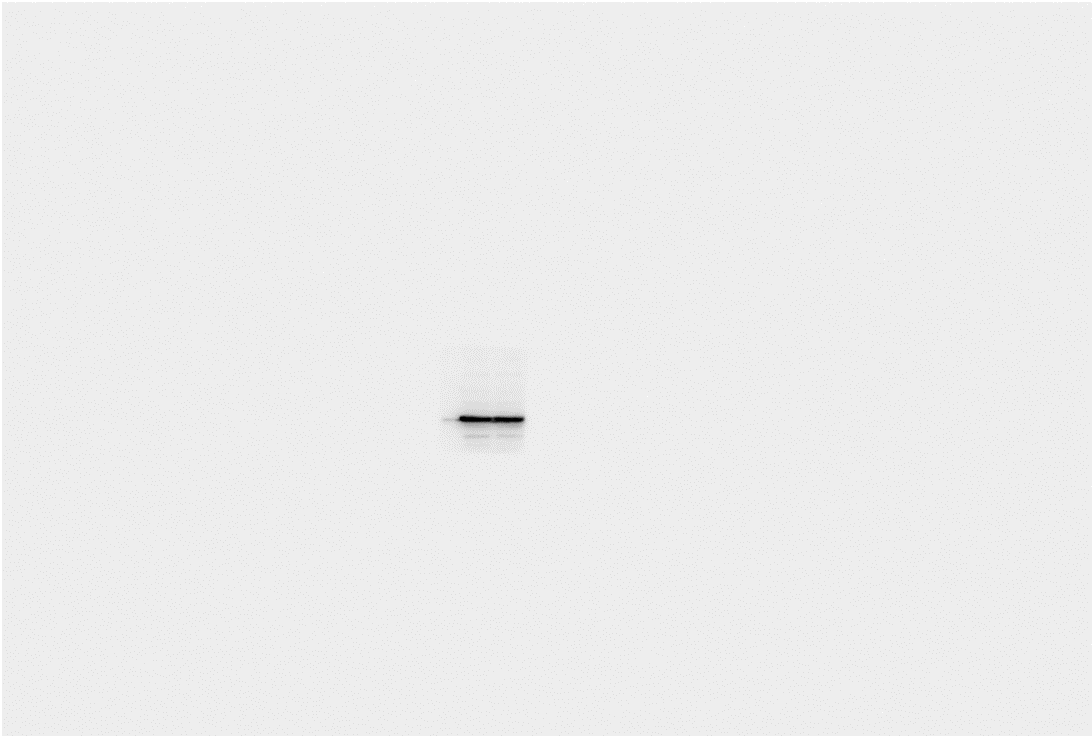


w.MDA-MB-231 P-JNK (CADM3)


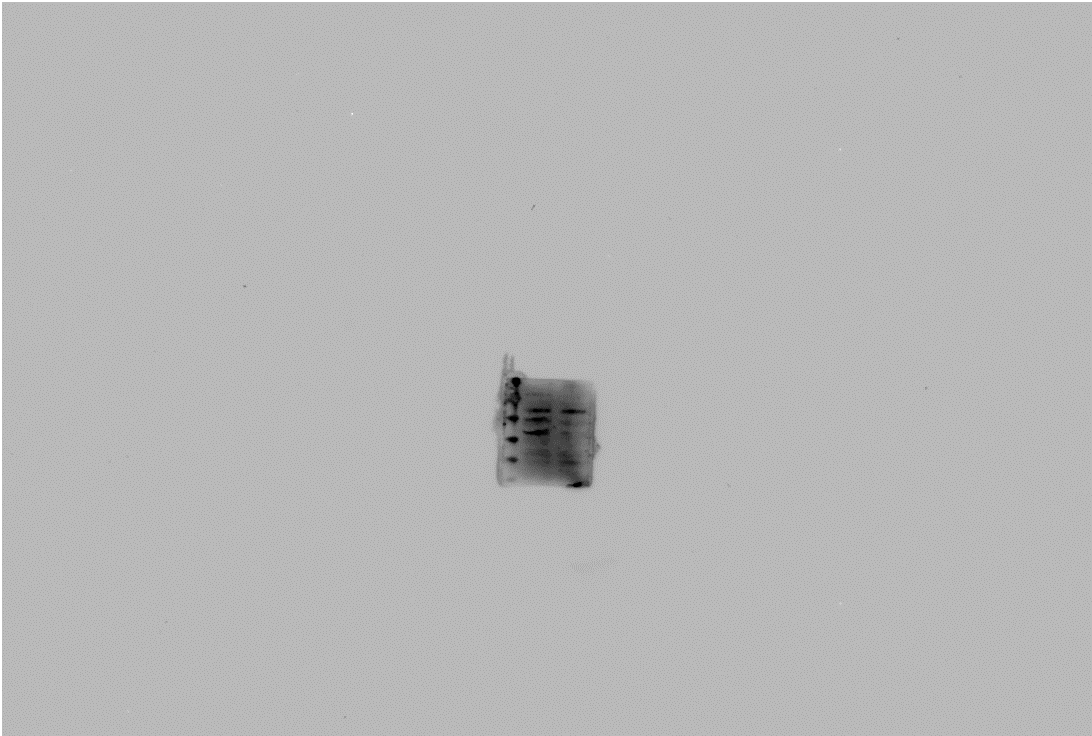


x. MDA-MB-231 P-JNK (GAPDH)


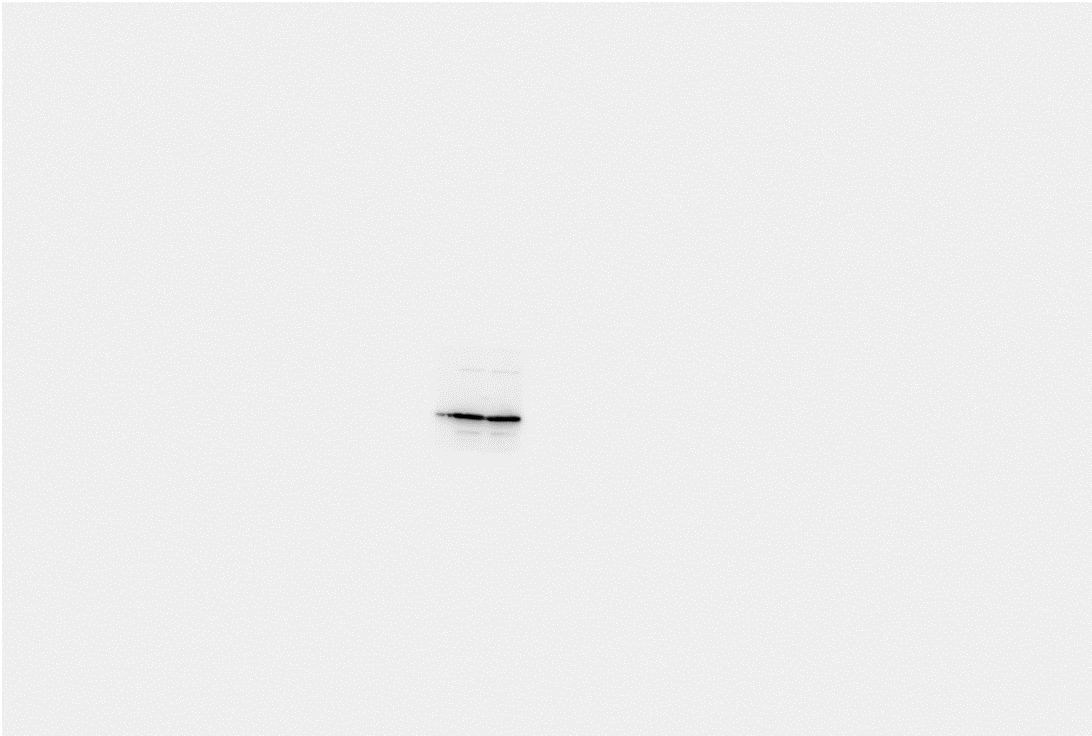


**Figure Legends**

**Supplementary figure.1** Protein expression of CADM3 in 8 cases of human BC and its adjacent pairs of normal tissues. (a-p): Full length gels and blots of figure 1E.

**Supplementary figure.2** The overexpression efficiency of CADM3 in MCF-7 and MDA-MB-231 cell lines verified by WB. (a-b): Full length gels and blots of figure 5A.

**Supplementary figure.3** The expressions of key proteins P38, ERK, JNK and their phosphorylated proteins in MAPK pathway were verified by WB assay. (a-x): Full length gels and blots of figure 8.
